# Hesperidin alleviates systemic inflammation and oxidative stress by remodeling adipose tissue lipid metabolism in periparturient dairy cows

**DOI:** 10.1186/s40104-026-01372-4

**Published:** 2026-04-05

**Authors:** Jian Tan, Ying Wang, Haoyu Niu, Luoyun Fang, Yuchao Zhao, Linshu Jiang

**Affiliations:** 1 Beijing Key Laboratory of Dairy Cow Nutrition, College of Animal Science and Technology, Beijing University of Agriculture, Beijing, 102206 China; 2 UCD School of Agriculture and Food Science, University College Dublin, Dublin, D04 V1W8 Ireland; 3 Key Laboratory of Molecular Animal Nutrition (Zhejiang University), Ministry of Education, Hangzhou, 310058 China

**Keywords:** Adipose tissue, Ceramides, Hesperidin, Lipidomics, Periparturient dairy cows, Sphingolipid metabolism

## Abstract

**Background:**

Periparturient dairy cows experience a pronounced negative energy balance that accelerates adipose tissue lipolysis and predisposes them to oxidative stress and inflammation. Plant flavonoids such as hesperidin are known for their antioxidant and immunomodulatory properties, yet their specific actions on adipose tissue metabolism during the transition period remain unclear. This study evaluated whether dietary hesperidin improves milk composition and systemic metabolic health while remodeling adipose tissue lipid metabolism in periparturient cows.

**Results:**

Hesperidin supplementation enhanced milk composition by increasing protein concentration and yield and lowering milk urea nitrogen, while dry matter intake and milk production were unaffected. In serum, hesperidin reduced non-esterified fatty acid, β-hydroxybutyrate, glucose, and insulin, and elevated adiponectin, leading to improved insulin sensitivity. Antioxidant capacity was strengthened, and several proinflammatory mediators, including IL-18, TNF-α, serum amyloid A, lipopolysaccharide-binding protein, caspase-1, and ASC, were significantly reduced. Three HES-derived metabolites (hesperetin-7-O-glucuronide, hesperetin, and hesperetin-7-O-sulfate) in serum and adipose tissue were detected. In adipose tissue, hesperidin increased total antioxidant capacity and superoxide dismutase activity and downregulated IL-18, NLRP3, and ASC, while adiponectin was elevated, indicating enhanced redox defenses and reduced inflammasome activation. Multi-omics analyses revealed consistent remodeling of adipose metabolism. Metabolomics and lipidomics showed decreased ceramides and acylcarnitines, together with increased sphingomyelins and glycerophospholipids, patterns that were also evident in serum lipid profiles. Proteomics further supported these findings by indicating upregulation of pathways related to fatty acid oxidation, mitochondrial function, and phospholipid and glutathione metabolism, alongside suppression of sphingolipid and innate immune signaling.

**Conclusions:**

Dietary hesperidin improved milk protein output and systemic metabolic health, enhanced antioxidant capacity, and alleviated inflammatory responses during the transition period. Integrated omics evidence indicates that hesperidin reprograms adipose lipid metabolism—particularly sphingolipid pathways—toward lower ceramide burden and higher sphingomyelin, while reinforcing mitochondrial and antioxidant functions. These adaptations highlight the potential of hesperidin as a nutritional strategy to improve adipose tissue function and whole-body metabolic homeostasis in periparturient cows.

**Supplementary Information:**

The online version contains supplementary material available at 10.1186/s40104-026-01372-4.

## Background

During the periparturient phase, dairy cows undergo substantial metabolic challenges, as the sharp rise in energy requirements for milk production often outpaces dry matter intake (DMI), leading to a negative energy balance (NEB) [1]. To compensate, body fat reserves are mobilized, but incomplete oxidation of fatty acids promotes ketone body formation. Such metabolic adjustments predispose animals to infectious diseases [2] and can aggravate the severity of mastitis [3]. In addition, NEB contributes to oxidative stress, defined as an imbalance between free radical generation and antioxidant defense, which further intensifies inflammatory responses [4, 5].

Recent reviews further emphasize that uncontrolled adipose tissue lipolysis not only generates excessive reactive oxygen species (ROS) but also initiates chronic inflammation and impairs insulin signaling, creating a vicious cycle that aggravates metabolic stress in periparturient cows [6, 7]. Excessive fat mobilization alters not only circulating non-esterified fatty acids (NEFA) and ketones but also the composition of lipid species within adipose tissue. For example, Zhao et al. [8] reported that cows experiencing high lipolysis postpartum exhibited elevated ceramide species and acylcarnitines, which were closely associated with reduced insulin sensitivity, increased oxidative stress, and impaired liver function. Recent multi-omics studies further highlight the central role of adipose tissue lipid metabolism in periparturient metabolic disorders. In cows with subclinical ketosis, adipose tissue exhibits profound disturbances in sphingolipid metabolism, characterized by accumulation of ceramides and sphingomyelins that correlate with impaired insulin sensitivity, enhanced lipolysis, and chronic inflammation [9]. Additionally, chronic nutrient surplus induces ceramide accumulation in multiple tissues, including adipose tissue, which in turn impairs insulin receptor expression and signaling, leading to diminished insulin sensitivity, and exacerbated metabolic inflammation in Holstein bulls [10]. Given adipose tissue’s central role in energy homeostasis and its endocrine function in regulating metabolism and immunity [11], there is growing interest in nutritional strategies that can temper excessive lipolysis and metabolic inflammation during this critical period.

In recent years, plant-derived bioactive compounds (phytonutrients or phytochemicals) have gained traction as dietary supplements to support metabolic health in transition dairy cows. Among phytochemicals, flavonoids, a diverse class of polyphenolic compounds abundant in fruits, herbs, and other plant sources, have received particular attention for use in ruminant diets [12]. Previous studies with polyphenol-rich extracts also indicate improved metabolic status, e.g., green tea and curcumin extracts reduced plasma NEFA and liver stress biomarkers in early-lactation cows [13], and grape seed extract lowered inflammatory acute-phase proteins in transition cows [14, 15]. Supplementation with plant polyphenol extracts containing green tea, capsicum, and fenugreek during heat stress not only improved dry matter intake, milk yield, and animal welfare but also enriched adipose tissue with proteins related to the Nrf2-mediated antioxidant response and acute phase signaling [16].

More recently, citrus peel extract (CPE), which is rich in flavonoids such as hesperidin and naringin, has been investigated as a potential nutritional strategy in dairy cows. Supplementation of CPE during the transition period improved rumen microbiota composition, reduced circulating NEFA and β-hydroxybutyrate (BHB), attenuated adipose tissue lipolysis and inflammation, and enhanced insulin sensitivity and liver metabolic function [17]. In lactating cows, citrus extract supplementation modified plasma fatty acid profiles and increased circulating insulin, suggesting systemic effects on lipid and glucose metabolism [18]. Furthermore, multi-omics analyses demonstrated that citrus flavonoid supplementation modulated sphingolipid metabolism, reducing ceramide accumulation and systemic inflammatory markers, thereby highlighting a link between citrus-derived flavonoids and lipid metabolism in lactating cows [19].

Taken together, these studies suggest that CPE and its bioactive flavonoids may exert beneficial effects on energy metabolism, oxidative stress, and lipid remodeling in dairy cows. However, whether specific citrus flavonoids such as hesperidin can directly regulate sphingolipid metabolism in adipose tissue during the periparturient period, which is a critical window for metabolic adaptation, remains unclear. This gap highlights the need to investigate the role of hesperidin in modulating adipose tissue lipid metabolism and systemic metabolic health in transition dairy cows. By integrating metabolomics, lipidomics, and proteomics, we aimed to characterize the effects of hesperidin on adipose tissue lipid remodeling, oxidative stress, and inflammatory responses, as well as its systemic impacts on metabolic health.

We hypothesized that hesperidin supplementation would attenuate excessive lipolysis and ceramide accumulation in adipose tissue, enhance antioxidant and anti-inflammatory capacity, and thereby improve overall metabolic adaptation during the periparturient period.

## Methods

### Design, treatments, and management

All animal use and handling protocols were reviewed and approved by the Institutional Animal Care and Use Committee of Beijing University of Agriculture (Protocol #BUA2023042-6).

A total of 32 multiparous Chinese Holstein cows (average BW: 694 ± 35 kg), in late gestation and entering their second to fourth lactations, were enrolled in a randomized complete block design. Blocking was performed according to parity, anticipated calving date, and body condition score (BCS). Sample size calculations indicated that 16 cows per treatment group would provide 80% power (α = 0.05) to detect at least a 30% change in serum cytokine concentrations, as suggested by prior work [20]. Animals within each block were randomly allocated to one of two diets: (1) a control group fed the basal total mixed ration (TMR) (CON), or (2) the basal TMR supplemented with 30 g/d of hesperidin (HES, purity ≥ 95%; Xi’an Xuquan Biotechnology Co., Ltd., Xi’an, China). Treatments were provided from 21 d before the expected calving date through 35 d of lactation. We chose to add 30 g/d per cow of hesperidin because a previous study we conducted on mid-lactation dairy cows showed that supplementing their diet with 100 g/d of citrus extract (containing approximately 20% hesperidin) significantly reduced pro-inflammatory cytokine levels and improved lactation performance [19].

Hesperidin was blended with 100 g of ground corn and offered once daily at 0700 h by top-dressing onto the TMR. Cows in the control group were provided the same amount of corn meal without additive as a placebo. During feeding, animals were restrained in head-locks, leaving one empty space between individuals, within a pen equipped with 70 head-lock positions. Each cow remained individually head-locked until the top-dress was completely consumed and was monitored throughout the entire process. About one week prior to the formal trial, a pretest was conducted in which cows were offered corn meal alone as a top-dress to evaluate the practicality of the feeding method. The test showed that each animal required roughly 15 s to finish the supplement, confirming the method’s consistency and suitability for use in the experiment.

Cows showing imminent calving signs were transferred to individual maternity pens and, if clinically normal, were moved into the lactation group within 24 h after parturition. From −50 to −22 d relative to expected calving, all animals received a far-off diet, followed by a close-up diet from −21 d until calving, and then a lactation diet through 35 DIM. Rations were formulated as TMR to meet or surpass nutrient requirements for dry and lactating cows based on NASEM [21]. Feed was provided twice daily at 0700 and 1500 h on an ad libitum basis during both prepartum and postpartum phases. Ingredient composition and nutrient analysis of the close-up and lactation rations are listed in Table S1 (Additional file 1). Daily feed allocation was adjusted to ensure approximately 5% refusals, and both refusals and actual intakes were recorded each day. Dry matter content of basal diet ingredients was determined weekly by oven-drying samples at 60 °C for 48 h, and individual weekly average DMI was subsequently calculated. Water was available ad libitum.

From calving until 5 weeks of lactation, cows were milked three times daily at 0600, 1400, and 2200 h. Milk yield was recorded at each milking, and composite milk samples were obtained weekly from all three milkings within a 24-h period. Samples were analyzed for fat, protein, lactose, somatic cell count (SCC), and milk urea nitrogen (MUN) using Fourier-transform infrared spectroscopy (LactoScope FTIR Advanced; Delta Instruments, Drachten, the Netherlands). Energy-corrected milk (ECM) output was estimated using the equation: ECM = (0.327 × milk yield) + (12.95 × fat yield) + (7.2 × protein yield). Fat-corrected milk standardized to 3.5% (3.5% FCM) was calculated as: 3.5% FCM = (0.432 × milk yield) + (16.23 × fat yield).

Cows were assessed daily for health by rectal thermometer, stethoscope, and physical examination by a veterinarian. Diseases recorded included displaced abomasum, ketosis, metritis, lameness, and “other digestive disorder” (diarrhea or hypermotile rumen). Ketosis was defined as having a plasma BHBA concentration > 1,400 μmol/L at any point during the experimental period. This cut-off value was adapted from Duffield [22] as a threshold for subclinical ketosis. Mastitis was defined as having a daily mean SCC > 400,000 cells/mL. Other diseases were diagnosed according to definitions established by Kelton et al. [23]. Disease incidences for control and HES cows, respectively, were retained placenta (2 vs. 1), milk fever (2 vs. 1), ketosis (1 vs. 0), metritis (0 vs. 1), displaced abomasum (0 vs. 0), mastitis (0 vs. 0), and lameness (0 vs. 0).

### Body weight, body condition, and energy balance

Body condition scores (1 to 5 scale) were assessed weekly by 2 individuals and BW was measured weekly from the preliminary period throughout the entire treatment period [24]. The BW and BCS were recorded always after milking but before feeding in the morning. On calving day, cows were weighed and their BCS was assessed after the collection of colostrum. Calf weights at birth were measured before the first colostrum was offered.

The energy balance (EB) was calculated according to NASEM [21] as follows:

$$\mathrm{NE}_\mathrm{L} \ \mathrm{intake} (\mathrm{NE}_\mathrm{L}\mathrm{I}) = \mathrm{DMI} \times \mathrm{NE}_\mathrm{L} \ \text{of the diet};$$

$$\mathrm{NE}_\mathrm{L}\mathrm{R} \ \mathrm{prepartum} = \mathrm{NE}_\mathrm{L}\ \mathrm{maintenance} + \mathrm{NE}_\mathrm{L}\ \mathrm{pregnancy};$$

$$\mathrm{NE}_\mathrm{L}\mathrm{R}\ \mathrm{postpartum} = \mathrm{NE}_\mathrm{L}\ \mathrm{maintenance} + \mathrm{NE}_\mathrm{L}\ \mathrm{milk};$$

$$\mathrm{EB}\ \mathrm{prepartum} = \mathrm{NE}_\mathrm{L}\mathrm{I} - \mathrm{NE}_\mathrm{L}\ \mathrm{maintenance} - \mathrm{NE}_\mathrm{L}\ \mathrm{pregnancy};$$

$$\mathrm{EB}\ \mathrm{postpartum} = \mathrm{NE}_\mathrm{L}\mathrm{I} - \mathrm{NE}_\mathrm{L}\ \mathrm{maintenance} - \mathrm{NE}_\mathrm{L} \mathrm{milk};$$

where NE_L_ maintenance, NE_L_ pregnancy, and NE_L_ milk were calculated as follows:

$$\mathrm{NE}_\mathrm{L}\ \mathrm{maintenance} = \left(\mathrm{BW}^{0.75} \times 0.10\right);$$

$$\begin{aligned} &\mathrm{NE}_\mathrm{L}\ \mathrm{pregnancy} = [(\text{Calf BW} \times 1.825) \times [0.0243 - (0.00000245 \times \text{day of gestation})] \times 4.16; \\&\text{and } \mathrm{NE}_\mathrm{L}\text{ milk} = \text{milk production} \times [(0.0929 \times \mathrm{fat}\%) + (0.055 \times \mathrm{protein}\%) + (0.0395 \times \mathrm{lactose}\%)].\end{aligned}$$

### Blood sampling and analysis

Blood was drawn from the coccygeal vein prior to the morning feeding, and serum was obtained by centrifugation at 3,000 × *g* for 10 min at 4 °C. Sampling was performed at d −21, −14, −7, −1 prepartum, and on d 3, 10, 20, and 30 postpartum. Serum obtained was stored at −80 °C until analysis. Serum concentrations of non-esterified fatty acids (NEFA; #BC0595), β-hydroxybutyrate (BHB; #BC5085), glucose (GLU; #BC2505), triglycerides (TG; #BC0625), total cholesterol (TC; #BC1985), total antioxidant capacity (T-AOC; #BC1315), and glutathione peroxidase (GSH-Px; #BC1195) were determined with commercial kits (Beijing Solarbio Science & Technology Co., Ltd., Beijing, China). The concentration of NEFA was quantified using a colorimetric method, in which NEFA react with a reagent to produce a colored complex measured at 550 nm; BHB was measured by an enzymatic assay, with the oxidation of BHB by β-hydroxybutyrate dehydrogenase resulting in a change in absorbance proportional to BHB concentration; GLU levels were determined using the glucose oxidase method, where glucose is oxidized to hydrogen peroxide, which reacts with a chromogen to produce a color change; TG concentration was measured enzymatically, where triglycerides are hydrolyzed by lipase to release glycerol, which reacts with a reagent to form a colored product measured at 510 nm; TC was assessed by colorimetric analysis, where cholesterol esters are hydrolyzed, and the free cholesterol reacts with a reagent to form a colored complex; T-AOC was determined by measuring the ability of antioxidants to reduce Fe^3+^-TPTZ to the blue Fe^2+^-TPTZ complex, with the intensity of the color change being directly proportional to antioxidant activity; and GSH-Px activity was quantified using a coupled enzyme reaction, where GSH-Px reduces hydrogen peroxide in the presence of reduced glutathione, and the reduction is measured by the change in absorbance at 340 nm. Levels of insulin (INS; #H203-1-1), adiponectin (#H179-1–2), IL-1β (#H002-1–2), IL-2 (#H003-1-1), IL-6 (#H007-1-2), IL-18 (#H015-1-2), TNF-α (#H052-1-2), serum amyloid A (SAA; #H134), lipopolysaccharide-binding protein (LBP; #H253-1-2), caspase-1 (Cas-1; #H074-1-2), and apoptosis-associated speck-like protein containing a CARD (ASC; #H597) were quantified using bovine-specific enzyme-linked immunosorbent assay (ELISA) kits (Nanjing Jiancheng Bioengineering Institute, Nanjing, China), based on antigen-antibody interactions and subsequent colorimetric detection. For all assays, intra- and inter-assay coefficients of variation were below 10%. All analyses were performed in triplicate. Detailed parameters for each ELISA kit are provided in Table S2 (Additional file 2).

### Adipose tissue collection and analysis

Subcutaneous adipose tissue was collected from the tail-head area of cows on d 5, 10, and 25 postpartum following the procedure outlined by Zhao et al. [9]. In brief, after administration of local anesthesia (15 mL of 2% lidocaine hydrochloride; VetTek) and aseptic preparation of the site with iodine scrub, iodine solution, and alcohol, a vertical skin incision of approximately 5 cm was made. The site of the incision was moved ventrally 3 to 4 cm at each collection time point. This anatomic location offers a wider surgical area for repeated AT sampling, it is less likely to have fecal contamination, and it is seldom used as an injection site. Around 5 g of adipose tissue was collected, flushed with physiological saline, mounted into screw-cup tubes, immediately frozen in liquid nitrogen, and kept at −80 °C until analysis. The incision site was sutured, disinfected, and then treated with an aluminum spray to provide a protective barrier.

The subcutaneous adipose tissue biopsy samples were washed with 1 mL of PBS and transferred to a homogenizing tube containing 1 mL of homogenizing solution (05892791001, Roche Diagnostics, Indianapolis, IN, USA) and homogenized. The homogenate was centrifuged at room temperature at 1,000 × *g* for 10 min. The clear homogenate below the upper fat layer was used for total protein concentration measurements, antioxidant status evaluation, and ELISA. The total protein concentration was measured by BCA method according to the manufacturer's protocol (#P0011; Beyotime Biotech Inc., Shanghai, China) in 3 replicates. T-AOC (#BC1315) and superoxide dismutase (SOD; #BC1315) activities in adipose tissue samples were assessed with commercial kits (Beijing Solarbio Science & Technology Co., Ltd., Beijing, China). The T-AOC assay is based on the ability of antioxidants in adipose tissue homogenates to reduce Fe^3+^-TPTZ to the blue Fe^2+^-TPTZ complex, whereas SOD activity was determined by its capacity to inhibit the reaction between superoxide anions and a chromogenic substrate. Adipose tissue concentrations of IL-1β (#H002-1-2), IL-6 (#H007-1-2), IL-18 (#H015-1-2), TNF-α (#H052-1-2), NOD-like receptor family pyrin domain-containing 3 (NLRP3; #H614-1-2), ASC (#H597), and adiponectin (#H179-1-2) were quantified using bovine-specific ELISA kits (Nanjing Jiancheng Bioengineering Institute, Nanjing, China) according to the manufacturer’s instructions. All measurements were performed in technical triplicate, and intra- and inter-assay coefficients of variation were consistently below 10%.

### Adipose tissue proteomics

Adipose tissue obtained on d 5 postpartum from 8 cows in the CON group and 8 cows in the HES group was used for proteomic, metabolomic, and lipidomic analyses. Samples preserved at −80 °C were thawed on ice and placed into MP homogenization tubes. Protein extraction was performed with lysis buffer (8 mol/L urea, 1% SDS, and protease inhibitors), followed by three rounds of high-speed homogenization (40 s each). The homogenates were then incubated on ice for 30 min with intermittent vortexing (every 5 min) and centrifuged at 12,000 × *g* for 30 min at 4 °C. Supernatants were collected, and protein concentration was quantified using the BCA assay (Thermo Fisher Scientific) according to the supplier’s protocol.

For digestion, 100 µg of protein from each sample was adjusted with lysis buffer, supplemented with 100 mmol/L triethylammonium bicarbonate (TEAB) and 10 mmol/L tris (2-carboxyethyl) phosphine (TCEP), and incubated at 37 °C for 60 min. Iodoacetamide (40 mmol/L) was added for alkylation in the dark at room temperature for 40 min. Proteins were precipitated with prechilled acetone (6:1, v/v; acetone:sample) at −20 ℃ for 4 h, pelleted by centrifugation (10,000 × *g*, 20 min), and resuspended in 100 µL 100 mmol/L TEAB. Digestion was performed overnight at 37 °C with trypsin at an enzyme-to-protein ratio of 1:50 (w/w).

Peptides were vacuum-dried, dissolved in 0.1% trifluoroacetic acid (TFA), purified using Oasis HLB cartridges (Waters, USA), and quantified with a peptide assay kit (Thermo Fisher Scientific, Cat. No. 23275). Data-independent acquisition (DIA) analysis was performed on a Vanquish Neo UHPLC system (Thermo) coupled to an Orbitrap Astral mass spectrometer (Thermo). Peptide separation was achieved on an ES906 column (150 µm × 15 cm, Thermo) under a 180-min gradient at a flow rate of 500 nL/min, using mobile phase A (2% acetonitrile and 0.1% formic acid in water) and mobile phase B (80% acetonitrile and 0.1% formic acid). The instrument was run in DIA mode with positive ion detection, a spray voltage of 1.5 kV, and a mass range of *m*/*z* 100–1700.

Raw DIA spectra were processed using Proteome Discoverer software (v 2.4, Thermo Fisher Scientific). Database search against a UniProt protein database of *Bos taurus* was performed according to the following parameters: (1) allowance of up to two missed cleavage sites for enzyme digestion; (2) a peptide length requirement of more than six amino acids; (3) the alkylation of cysteine (C) as a fixed modification; (4) variable modifications, including oxidation, acetylation, and deamination of methionine (M); and (5) the utilization of razor and unique peptides for protein quantification. The false discovery rate (FDR) for peptide identification was calculated using the Percolator algorithm in the Proteome Discoverer workflow based on the search results against a decoy database and was set at 1% FDR. However, at least 2 unique peptides and 5% FDR were required for reporting confidently identified proteins. Protein quantification was accomplished by correlating the relative intensities of reporter ions extracted from tandem mass spectra to that of the peptides selected for MS/MS fragmentation. Statistical analysis was conducted in R (v 4.3.2) with Student’s *t*-test, and proteins were considered differentially expressed when fold change exceeded 2 and the adjusted *P*-value (FDR corrected) was < 0.05.

### Adipose tissue untargeted metabolomics

Around 50 mg of adipose tissue was transferred into a 2-mL microcentrifuge tube with a 6-mm stainless steel bead. Metabolites were extracted using 400 μL of methanol–water solution (4:1, v/v) containing 0.02 mg/mL L-2-chlorophenylalanine as an internal standard. Samples were homogenized at −10 °C and 50 Hz for 6 min with a tissue grinder, followed by ultrasonic treatment for 30 min at 5 °C and 40 kHz. The mixture was then kept at −20 °C for 30 min and centrifuged at 13,000 × *g* for 15 min at 4 °C. The resulting supernatant was transferred into autosampler vials with inserts for LC–MS/MS analysis.

Chromatographic separation and mass spectrometric analysis were conducted using a UHPLC–Q Exactive HF-X platform (Thermo Fisher Scientific, Waltham, MA, USA) at Shanghai Majorbio Bio-Pharm Technology Co., Ltd. Samples were injected (2 μL) onto an Accucore C30 column (100 mm × 2.1 mm, 2.6 μm; Thermo Fisher Scientific). The mobile phases consisted of solvent A (10 mmol/L ammonium acetate with 0.1% formic acid in water) and solvent B (acetonitrile with 0.1% formic acid). The gradient program was set as follows: 0–1 min, 5% B; 1–11 min, linear increase to 95% B; 11–19.5 min, return to 5% B.

Raw LC–MS data were processed using Progenesis QI software (Waters, UK) for baseline correction, peak alignment, noise filtering, and normalization. Metabolites were annotated by comparing accurate mass, retention time, and MS/MS fragmentation spectra with entries in public databases, including HMDB, METLIN, and KEGG.

### Adipose tissue and serum untargeted lipidomics

For untargeted lipidomics, serum collected on d 10 postpartum was analyzed from 8 cows in the CON group and 8 in the HES group. Serum lipids were isolated using the procedure of Draijer et al. [25], whereas adipose lipids were extracted according to Lam et al. [26]. For adipose tissue, ~50 mg of frozen sample was placed in a 2-mL tube with a 6-mm bead, mixed with 280 μL methanol:water (2:5, v/v) and 400 μL MTBE, homogenized at −10 °C (50 Hz, 6 min), sonicated for 30 min (5 °C, 40 kHz), and kept at −20 °C for 30 min. After centrifugation (13,000 × *g*, 15 min, 4 °C), 350 μL of the upper phase was dried under nitrogen and re-dissolved in 100 μL isopropanol:acetonitrile (1:1, v/v). The solution was vortexed, briefly sonicated (ice bath, 5 min), centrifuged (13,000 × *g*, 10 min, 4 °C), and the supernatant transferred into autosampler vials.

For serum extraction, 100 μL of sample was combined with 300 μL methanol and 400 μL MTBE, vortexed, and incubated at 4 °C for 10 min. The mixture was then sonicated (5 °C, 40 kHz, 20 min) and centrifuged (13,000 × *g*, 15 min, 4 °C). The supernatant was evaporated under nitrogen, reconstituted in 100 μL isopropanol:acetonitrile (1:1, v/v), and prepared for LC–MS/MS analysis.

Lipidomic analysis was conducted on a UHPLC system (Vanquish; Thermo Fisher Scientific, USA) coupled to a Q Exactive Plus Orbitrap mass spectrometer (Thermo Fisher Scientific, USA). Separation was achieved on a Hypersil GOLD C18 column (100 mm × 2.1 mm, 1.9 μm). The mobile phases consisted of solvent A (acetonitrile:water, 60:40, v/v) and solvent B (isopropanol:acetonitrile, 90:10, v/v), both supplemented with 10 mmol/L ammonium formate and 0.1% formic acid. A constant flow rate of 0.3 mL/min was used. The gradient program started at 30% B (0–2 min), increased to 100% B from 2 to 25 min, maintained at 100% B for 5 min, then returned to 30% B at 30.1 min, followed by equilibration until 35 min. Lipid identification and quantification were performed using LipidSearch software (version 4.2; Thermo Fisher Scientific, USA), based on accurate mass, retention time, and MS/MS fragmentation spectra.

### Targeted analysis of hesperidin metabolites in adipose tissue and serum

To analyze the targeted metabolites of hesperidin in both serum and adipose tissue, LC–MS was employed. Serum samples (200 µL) were mixed with 800 µL of ice-cold methanol, vortexed, and centrifuged at 10,000 × *g* for 10 min at 4 °C. The supernatant was collected, dried under nitrogen, and reconstituted in 200 µL of methanol:water (80:20, v/v). Adipose tissue (100 mg) was homogenized in 1 mL methanol, centrifuged, and the supernatant was dried under nitrogen and reconstituted similarly. Chromatographic separation was performed on an ACQUITY UPLC system (Waters, Milford, MA, USA) equipped with a BEH C18 column (2.1 mm × 100 mm, 1.7 µm, Waters, USA). The mobile phase consisted of solvent A (0.1% formic acid in water) and solvent B (0.1% formic acid in acetonitrile). Chromatographic separation was performed using a gradient elution program at a flow rate of 0.3 mL/min. The gradient was set as follows: 5% B from 0 to 2 min, increased to 25% B at 7 min, then to 50% B at 11 min, followed by an increase to 95% B from 12 to 14 min. The system was subsequently returned to the initial conditions (5% B) and equilibrated for 4 min prior to the next injection. The column temperature was maintained at 40 °C, and the injection volume was 5 µL.

Mass spectrometric detection was carried out on a QTRAP 5500 triple quadrupole mass spectrometer (AB Sciex, Framingham, MA, USA) equipped with an electrospray ionization (ESI) source operating in both positive and negative ion modes. Multiple reaction monitoring (MRM) was employed for quantification of hesperidin, hesperetin, hesperetin-7-O-glucoside, and hesperetin-7-O-sulfate. Metabolite quantification was performed using a standard curve for each compound prepared from synthetic standards. The results were expressed as ng/mL for serum and ng/mg tissue for adipose tissue. Quality control samples, consisting of pooled serum and adipose tissue, were analyzed every 10 samples to ensure consistency and precision, with all assays performed in triplicate for reproducibility.

### Statistical analysis

Statistical analyses for DMI, milk production traits, serum biomarkers, and adipose tissue measurements were performed with SAS software (version 9.4; SAS Institute Inc., Cary, NC, USA). The data were evaluated using the following statistical model:

$$Y_{ijkl}=\mu+ T_i+P_j+TP_{ij}+B_k+C(B)_{l(k)}+e_{ijkl},$$

where *Y*_*ijkl*_ = observations for dependent variables; *µ* = overall mean, *T*_*i*_ = fixed effect of treatment (CON or HES); *P*_*j*_ = fixed effect of time period (day or week) as a repeated measure; *TP*_*ij*_ = fixed effect of treatment and time period interaction; *B*_*k*_ = fixed effect of block; *C*(*B*)_*l*(*k*)_ = random effect of cow nested in block; and *e*_*ikl*_ or *e*_*ijkl*_ = residuals. Previous 305-d mature-equivalent milk yield values and pretreatment (collected during the week before assignment to treatment) values for DMI, serum variables, BW, and BCS were used as covariates during their respective analyses.

Time relative to calving was treated as the repeated measure, and eight covariance structures were evaluated; the structure yielding the lowest Akaike information criterion was applied for each trait. To control type I error, the Tukey–Kramer adjustment was used with Kenward–Roger corrected degrees of freedom. Residuals were checked for normal distribution, and alternative transformations (e.g., log-normal) were applied when necessary. Results were expressed as least squares means (LSM), with statistical significance declared at *P* ≤ 0.05 and tendencies noted when 0.05 < *P* ≤ 0.10.

For untargeted metabolomic and lipidomic data, principal component analysis (PCA) and orthogonal partial least squares–discriminant analysis (OPLS-DA) were applied to assess clustering patterns and highlight discriminant features. Variable importance in projection (VIP) values were derived from OPLS-DA, and features with VIP > 1.5 and *P* < 0.05 (Student’s* t*-test, FDR corrected) were defined as significantly different between treatments. Pathway enrichment of differential metabolites and lipids was conducted in MetaboAnalyst 6.0, using default settings and the Bos taurus pathway library [27].

## Results

### DMI, BW, BCS, and EB

**Fig. 1:**
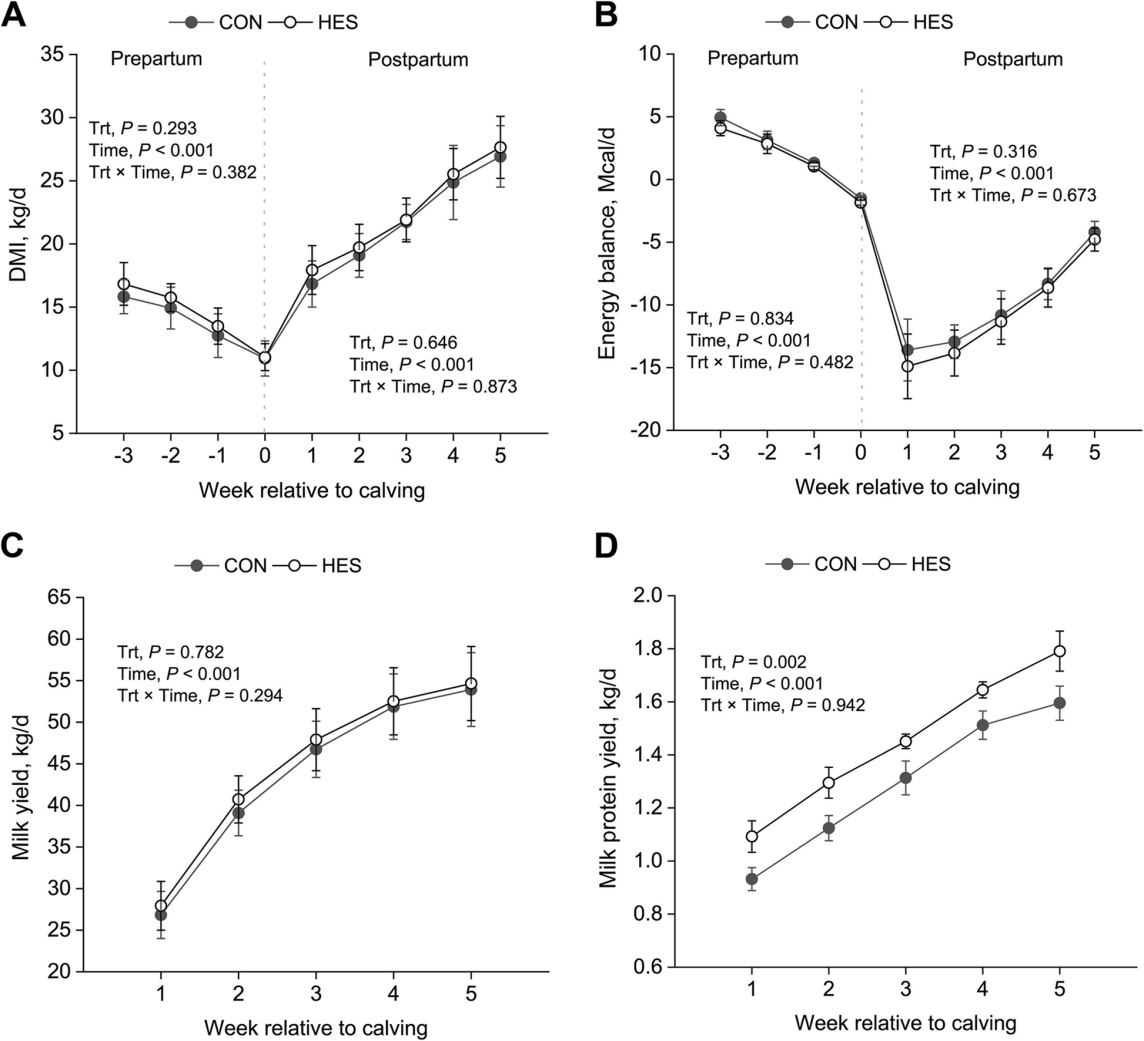
Changes on periparturient (**A**) dry matter intake, (**B**) energy balance, (**C**) milk yield, and (**D**) milk protein yield in cows fed with hesperidin. A total of 32 cows were assigned to 1 of 2 dietary treatments (TMR with or without HES) in a randomized complete block design. Serum indices were measured on d −21, −14, −7, −1, 3, 10, 20, and 30 relative to calving. CON, no supplemental hesperidin; HES, 30 g/d hesperidin; Trt, treatment. Daily results were averaged into weekly means before data analyses were conducted. Error bars represent SE of the LSM

Main effects and interactions for prepartal and postpartal DMI, BW, BCS, and EB are presented in Table 1. Neither prepartal nor postpartal DMI, BW, BW change, BCS, BCS change, and EB were affected (*P* > 0.05) by dietary treatments. No interaction was observed (*P* > 0.05) on DMI (Fig. 1A), BW, BCS, and EB (Fig. 1B) between HES and time.

**Table 1:** The effects of supplementation of hesperidin on dry matter intake, bodyweight, body condition score, and energy balance of dairy cows

Item^1^	Treatment^2^		SEM	*P*-value^3^		
	CON	HES		Trt	Time	Trt × Time
Prepartum^4^						
DMI, kg/d	13.5	14.2	0.42	0.293	<0.001	0.382
BW, kg	762	776	15.3	0.623	0.041	0.605
BW change, kg	19.5	20.2	3.12	0.821	—	—
BCS	3.38	3.43	0.031	0.525	0.114	0.746
BCS change	−0.09	−0.15	0.042	0.482	—	—
Energy balance, Mcal/d	3.12	2.66	0.380	0.834	<0.001	0.482
Postpartum^5^						
DMI, kg/d	21.9	22.2	0.59	0.646	<0.001	0.873
BW, kg	701	699	8.40	0.832	0.028	0.517
BW change, kg	−44.3	−45.8	7.39	0.306	—	—
BCS	3.26	3.29	0.040	0.428	0.043	0.422
BCS change	−0.23	−0.25	0.092	0.940	—	—
Energy balance, Mcal/d	−9.98	−10.69	0.454	0.316	<0.001	0.673

^1^*DMI* Dry matter intake, *BW* Body weight, *BCS* Body condition score

^2^CON, no supplemental hesperidin; HES, 30 g/d hesperidin

^3^Trt = treatment

^4^Prepartum parameters were analyzed from −21 d to calving (*n* = 16)

^5^Postpartum parameters were analyzed from calving to 35 DIM (*n* = 16)

### Milk production and composition

The yields of milk and protein are shown in Table 2 and Fig. 1C–D. Additionally, the yields of ECM, 3.5% FCM, and milk composition contents are presented in Table 2. There were time effects for milk yield (*P* < 0.001; Fig. 1C), 3.5% FCM (*P* = 0.023), and ECM (*P* < 0.001); however, no treatment effects or treatment and time interactions were detected during the postpartum period. Regarding milk composition, HES supplementation increased milk protein concentration (*P* = 0.008) and protein yield (*P* = 0.002; Fig. 1D) compared with CON, while milk fat percentage, fat yield, lactose percentage, lactose yield, and SCC were unaffected (*P* > 0.05). MUN was lower in the HES group (*P* = 0.013), and a significant treatment × time interaction was observed for MUN (*P* = 0.023). Feed efficiency indices, including milk yield/DMI, 3.5% FCM/DMI, and ECM/DMI, did not differ between treatments (*P* > 0.05).

**Table 2:** The effects of supplementation of hesperidin on production performance of dairy cows

Item^1^	Treatment^2^		SEM	*P*-value^3^		
	CON	HES		Trt	Time	Trt × Time
Milk yield, kg/d	43.7	44.7	1.63	0.782	<0.001	0.294
3.5% FCM^4^, kg/d	46.8	47.7	1.50	0.515	0.023	0.790
ECM^5^, kg/d	45.9	47.6	1.64	0.621	<0.001	0.383
Milk composition						
Fat, %	3.93	3.91	0.092	0.940	<0.001	0.378
Fat, kg/d	1.72	1.75	0.024	0.731	0.010	0.344
Protein, %	2.98	3.24	0.028	0.008	<0.001	0.646
Protein, kg/d	1.30	1.45	0.020	0.002	<0.001	0.942
Lactose, %	5.19	5.17	0.043	0.832	0.105	0.568
Lactose, kg/d	2.26	2.31	0.032	0.824	<0.001	0.757
MUN, mg/dL	13.1	10.7	0.53	0.013	0.007	0.023
SCC, ×10^3^ cells/mL	167	132	16.8	0.240	0.062	0.158
Feed efficiency						
Milk yield/DMI	2.00	2.01	0.041	0.383	0.839	0.632
3.5%FCM/DMI	2.14	2.15	0.023	0.567	0.072	0.590
ECM/DMI	2.10	2.14	0.036	0.451	0.038	0.787

^1^*FCM* Fat corrected milk, *ECM* Energy corrected milk, *MUN* Milk urea nitrogen, *SCC* Somatic cell count

^2^CON, no supplemental hesperidin; HES, 30 g/d hesperidin. Milk samples were collected from cows weekly (*n* = 16)

^3^Trt, treatment

^4^ECM was calculated as: ECM = (0.327 × milk yield) + (12.95 × fat yield) + (7.2 × protein yield)

^5^3.5% FCM was calculated as: 3.5% FCM = (0.432 × milk yield) + (16.23 × fat yield)

### Serum metabolic parameters

**Fig. 2:**
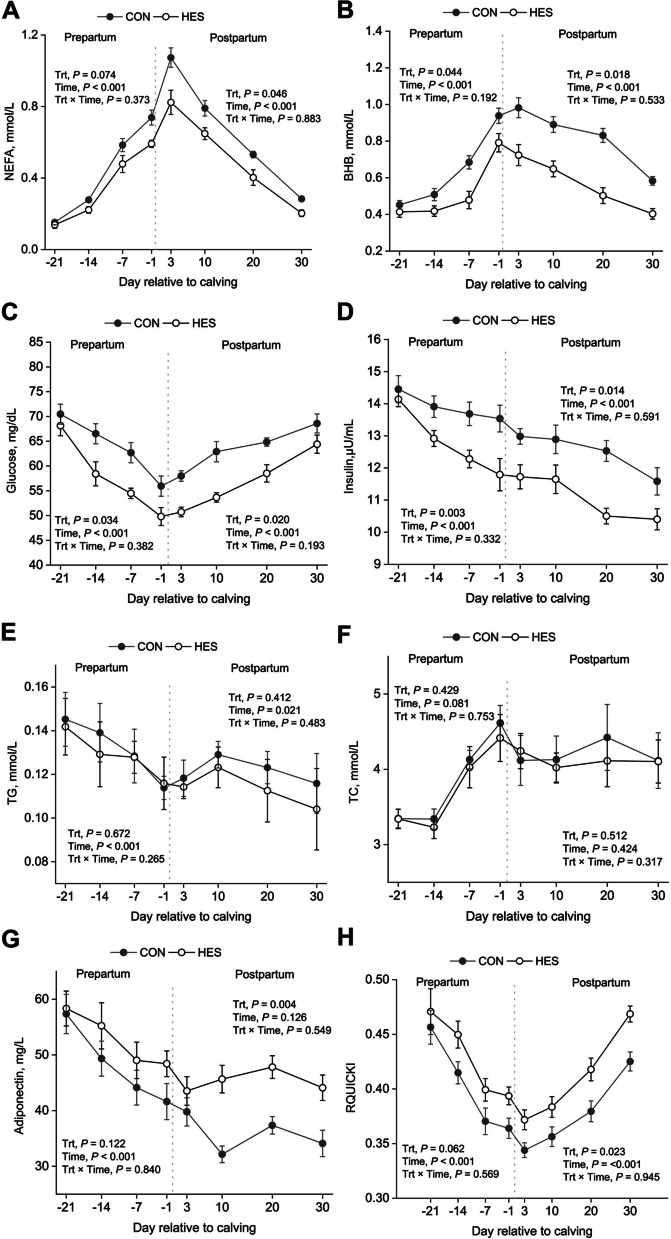
Changes on serum glucose and lipid metabolism indices in periparturient cows fed with hesperidin. **A** NEFA; **B** BHB; **C** glucose; **D** insulin; **E** TG; **F** TC; **G** adiponectin; **H** RQUCKI. A total of 32 cows were assigned to 1 of 2 dietary treatments (TMR with or without HES) in a randomized complete block design. Serum indices were measured on d −21, −14, −7, −1, 3, 10, 20, and 30 relative to calving. CON, no supplemental hesperidin; HES, 30 g/d hesperidin; Trt, treatment; NEFA, non-esterified fatty acid; BHB, β-hydroxybutyrate; TG, triglyceride; TC, total cholesterol; RQUICKI, revised quantitative insulin sensitivity check index. Error bars represent SE of the LSM

Serum indices of glucose and lipid metabolism are presented in Table 3 and Figs. 1 and 2. Sampling time exerted significant effects on nearly all variables (*P* < 0.05), but no treatment × time interactions were detected. Postpartum serum NEFA concentration was lower (*P* < 0.05) for cows fed HES vs. CON, and tended (*P* = 0.074) to be lower for HES vs. CON (Fig. 2A). Cows fed HES have lower concentrations of serum BHB (Fig. 2B), glucose (Fig. 2C), and insulin (Fig. 2D) during the prepartum period (*P* < 0.05) and postpartum period (*P* < 0.05). Serum TG (Fig. 2E) and TC (Fig. 2F) were not affected by treatment during either the prepartum (*P* > 0.10) or postpartum (*P* > 0.10) period. The HES treatment did not affect serum adiponectin concentration relative to the CON group during the prepartum period (*P* = 0.122), but it increased adiponectin after calving (Fig. 2G). A tendency (*P* = 0.062) for greater revised quantitative insulin sensitivity check index (RQUCKI) was observed in HES cows than in CON during the prepartum period (Fig. 2H), whereas HES addition resulted in greater RQUICKI in HES cows than CON during the postpartum period (*P* = 0.023).

**Table 3:** The effects of supplementation of hesperidin on serum glucose and lipid metabolism indices

Item^1^	Treatment^2^		SEM	*P*-value^3^		
	CON	HES		Trt	Time	Trt × Time
Prepartum						
NEFA, mmol/L	0.438	0.357	0.0162	0.074	<0.001	0.373
BHB, mmol/L	0.646	0.525	0.0382	0.044	<0.001	0.192
Glucose, mg/dL	63.9	57.7	1.82	0.034	<0.001	0.382
Insulin, μU/mL	13.9	12.8	0.31	0.003	<0.001	0.332
TG, mmol/L	0.132	0.129	0.0104	0.672	<0.001	0.265
TC, mmol/L	3.86	3.75	0.216	0.429	0.081	0.753
Adiponectin, mg/L	48.1	52.8	2.92	0.122	<0.001	0.840
RQUICKI^4^	0.401	0.428	0.0079	0.062	<0.001	0.569
Postpartum						
NEFA, mmol/L	0.670	0.519	0.0325	0.046	<0.001	0.883
BHB, mmol/L	0.822	0.569	0.0398	0.018	<0.001	0.533
Glucose, mg/dL	63.7	56.8	1.37	0.020	<0.001	0.193
Insulin, μU/mL	12.5	11.1	0.35	0.014	<0.001	0.591
TG, mmol/L	0.122	0.114	0.0119	0.412	0.021	0.483
TC, mmol/L	4.20	4.12	0.264	0.512	0.424	0.317
Adiponectin, mg/L	35.9	45.3	1.99	0.004	0.126	0.549
RQUICKI	0.376	0.410	0.0068	0.023	<0.001	0.945

^1^*NEFA* Non-esterified fatty acid, *BHB* β-Hydroxybutyrate, *TG* Triglyceride, *TC* Total cholesterol, *RQUICKI* Revised quantitative insulin sensitivity check index

^2^CON, no supplemental hesperidin; HES, 30 g/d hesperidin. Serum indices were measured on d −28, d−14, −7, −1, 3, 10, 20, and 30 relative to calving (*n* = 16)

^3^Trt, treatment

^4^The revised quantitative insulin sensitivity check index (RQUICKI; Holtenius and Holtenius, 2007) was calculated as follows: RQUICKI = 1/[log(serum glucose mg/dL) + log(serum insulin μU/mL) + log(serum NEFA mmol/L)]. Lower RQUICKI values are indicative of reduced insulin sensitivity

Serum antioxidant capacity and inflammatory markers are summarized in Table 4 and Fig. S1–S3 (Additional file 3). HES supplementation significantly enhanced T-AOC during the prepartum (*P* = 0.014) and postpartum period (*P* = 0.004) (Fig. S1A), while GSH-Px activity was not affected (*P* > 0.50) during either the prepartum or postpartum period (Fig. S1B). During both the prepartum and postpartum period, serum IL-1β (Fig. S2A), IL-18 (Fig. S2B), Cas-1 (Fig. S2C), and ASC (Fig. S2D) concentrations were lower (*P* < 0.05) in cows fed HES compared with CON. We observed a treatment (Trt) × time interaction in serum ASC during the prepartum (*P* = 0.016) and postpartum period (*P* = 0.021). The interaction was primarily driven by lower ASC concentrations (*P* < 0.01) in HES cows compared to controls on d −7, −1, and 30 relative to calving. HES supplementation did not affect (*P* > 0.10) serum IL-2 (Fig. S2E) or IL-6 (Fig. S2F) during either the prepartum or postpartum period, whereas lower concentration of prepartum and postpartum TNF-α (*P* < 0.05) was observed in HES cows than CON (Fig. S2G). Compared with control, cows supplemented with HES had lower serum concentration of SAA during the prepartum (*P* = 0.003) and postpartum period (*P* < 0.001) (Fig. S3A). Prepartum concentration of serum LBP tended (*P* = 0.063) to be lower for HES vs. CON (Fig. S3B), while postpartum LBP was significantly lower (*P* = 0.005) for cows fed HES vs. CON.

**Table 4:** The effects of supplementation of hesperidin on serum antioxidant status and proinflammatory factors

Item^1^	Treatment^2^		SEM	*P*-value^3^		
	CON	HES		Trt	Time	Trt × Time
Prepartum						
T-AOC, U/L	0.161	0.201	0.014	0.014	<0.001	0.352
GSH-Px, U/mL	205	207	2.3	0.548	0.062	0.346
IL-1β, ng/L	86.6	81.3	1.67	0.018	0.550	0.731
IL-2, ng/L	290	295	1.93	0.148	<0.001	0.596
IL-6, ng/L	136	133	1.8	0.739	<0.001	0.207
IL-18, ng/L	28.6	23.5	0.54	0.019	0.041	0.488
TNF-α, ng/L	33.6	29.8	1.03	0.027	0.021	0.703
SAA, ng/mL	5.90	4.85	0.125	0.003	<0.001	0.837
LBP, ng/mL	55.6	53.8	0.73	0.063	0.237	0.545
Cas-1, pg/mL	22.6	20.2	0.43	0.015	0.030	0.348
ASC, pg/mL	13.3	10.7	0.29	0.021	0.012	0.035
Postpartum						
T-AOC, U/L	0.150	0.209	0.015	0.004	<0.001	0.627
GSH-Px, U/mL	205	208	2.3	0.730	<0.001	0.569
IL-1β, ng/L	89.1	78.3	1.72	<0.001	0.684	0.799
IL-2, ng/L	275	274	2.40	0.303	<0.001	0.058
IL-6, ng/L	136	132	2.3	0.625	<0.001	0.830
IL-18, ng/L	20.1	14.1	1.21	0.007	0.014	0.736
TNF-α, ng/L	31.3	25.6	1.26	0.002	<0.001	0.248
SAA, ng/mL	5.32	3.79	0.144	<0.001	<0.001	0.860
LBP, ng/mL	64.1	58.0	1.13	0.005	<0.001	0.649
Cas-1, pg/mL	15.9	13.0	0.42	0.010	<0.001	0.073
ASC, pg/mL	10.0	8.94	0.30	0.016	<0.001	0.042

^1^*T-AOC* Total antioxidant capacity, *GSH-Px* Glutathione peroxidase, *IL* Interleukin, *TNF-α* Tumor necrosis factor α, *SAA* Serum amyloid A, *LBP* Lipopolysaccharide binding protein, *Cas-1* Caspase-1, *ASC* Apoptosis-associated speck-like protein containing a CARD

^2^CON, no supplemental hesperidin; HES, 30 g/d hesperidin. Serum indices were measured on d −21, −14, −7, −1, 3, 10, 20, and 30 relative to calving (*n* = 16)

^3^Trt, treatment

### Adipose tissue metabolic parameters

**Fig. 3:**
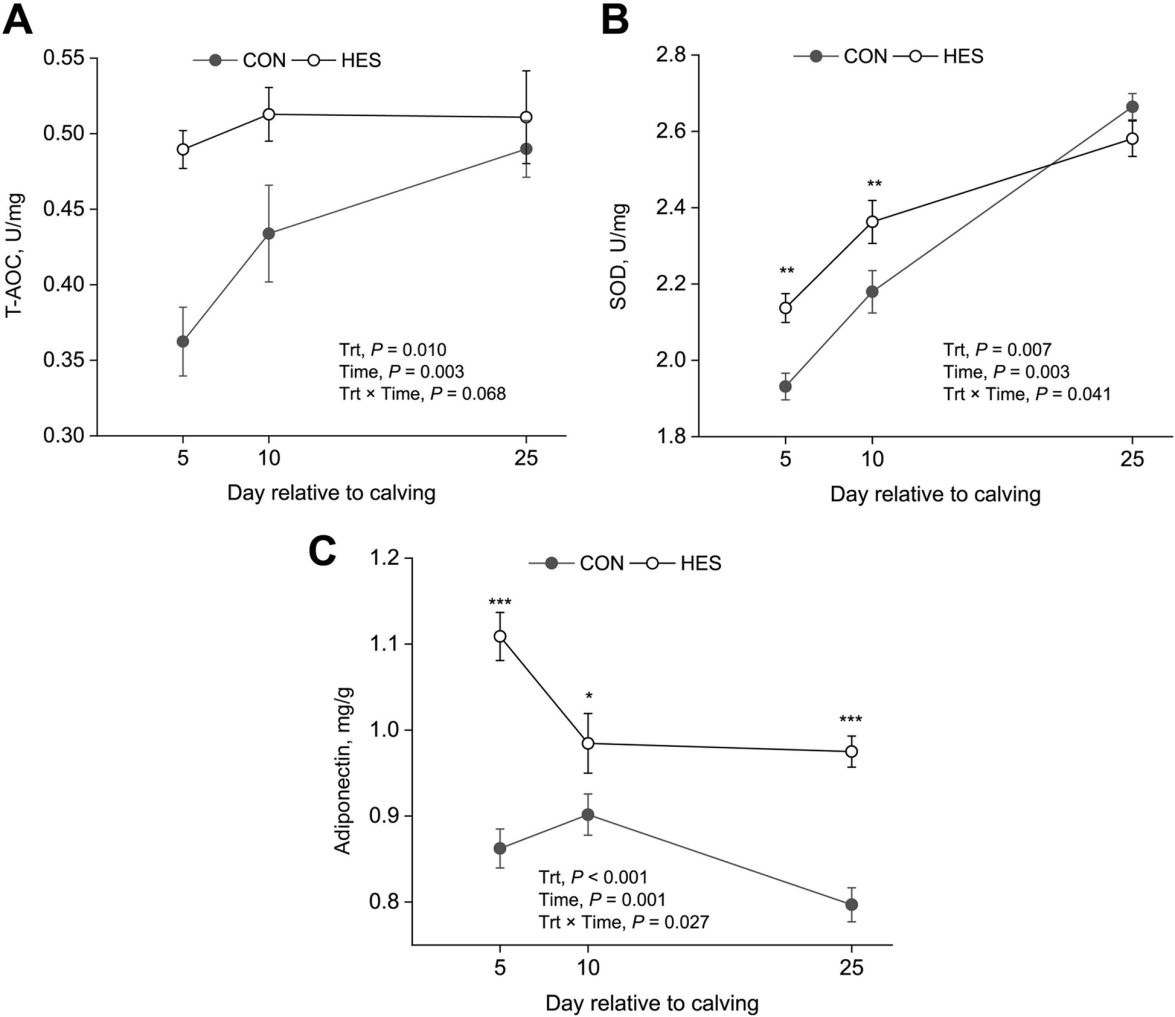
Changes on adipose tissue antioxidant status and adiponectin in periparturient cows fed with hesperidin. **A** T-AOC; **B** SOD; **C** adiponectin. A total of 32 cows were assigned to 1 of 2 dietary treatments (TMR with or without HES) in a randomized complete block design. Adipose tissue was biopsied on d 5, 10, and 25 relative to calving. CON, no supplemental hesperidin; HES, 30 g/d hesperidin; Trt, treatment; T-AOC, total antioxidant capacity; SOD, superoxide dismutase. Error bars represent SE of the LSM. If interactions between treatment and time were significant (*P* < 0.05), data were analyzed individually for each time point using *t*-test (^*^P < 0.05; ^*^^*^P < 0.01; ^***^P < 0.001)

**Fig. 4:**
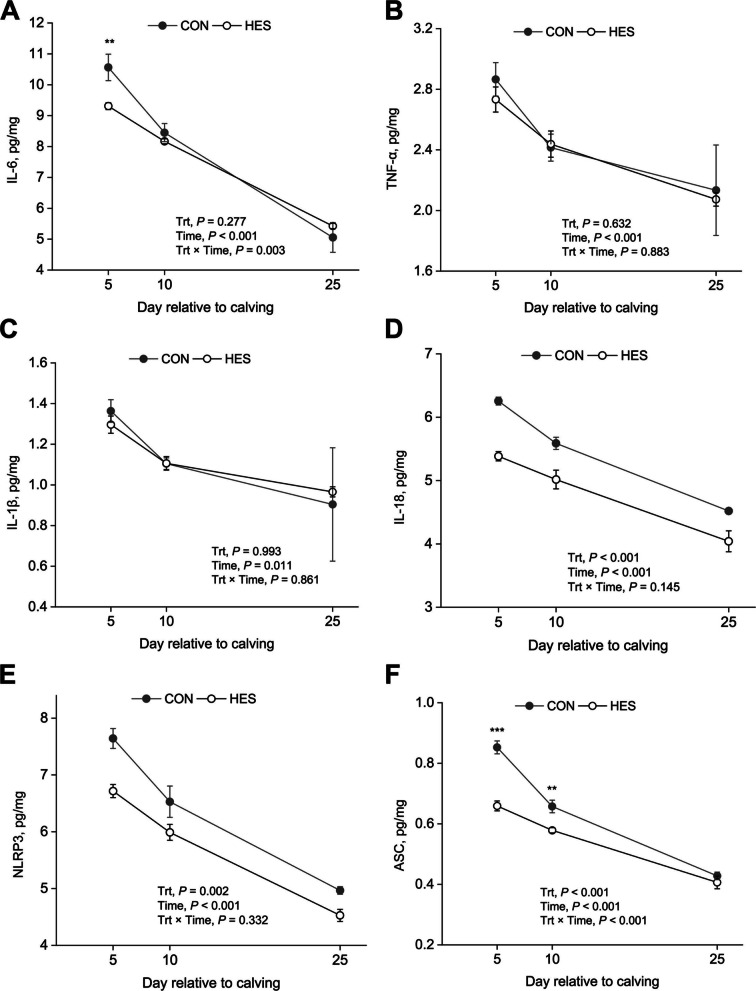
Changes on adipose tissue NLRP3 inflammasome and proinflammatory cytokines in periparturient cows fed with hesperidin. **A** IL-6; **B** TNF-α; **C** IL-1β; **D** IL-18; **E** NLRP3; **F** ASC. A total of 32 cows were assigned to 1 of 2 dietary treatments (TMR with or without HES) in a randomized complete block design. Adipose tissue was biopsied on d 5, 10, and 25 relative to calving. CON, no supplemental hesperidin; HES, 30 g/d hesperidin; Trt, treatment; IL, interleukin, TNF-α, tumor necrosis factor α; NLRP3, NOD-like receptor thermal protein domain associated protein 3; ASC, apoptosis-associated speck-like protein containing a CARD. Error bars represent SE of the LSM. If interactions between treatment and time were significant (*P* < 0.05), data were analyzed individually for each time point using *t*-test (^*^P < 0.05; ^*^^*^P < 0.01; ^***^P<0.001)

Postpartum biochemical parameters of adipose tissue are presented in Table 5 and Figs. 3 and 4. Supplementation with HES markedly enhanced the antioxidant profile, as evidenced by higher T-AOC (*P* = 0.010; Fig. 3A) and SOD activity (*P* = 0.007; Fig. 3B) relative to the control. We detected a Trt × time interaction (*P* = 0.041) on SOD activity. The Trt × time observed in SOD was associated with a greater (*P* < 0.05) activity in HES cows than control at 5 and 10 DIM. Adiponectin level in adipose tissue were elevated in response to hesperidin (*P* < 0.05) at 5, 10, and 25 DIM (Fig. 3C).

**Table 5:** The effects of supplementation of hesperidin on biochemical parameters in adipose tissue

Item^1^	Treatment^2^		SEM	*P*-value^3^		
	CON	HES		Trt	Time	Trt × Time
T-AOC, U/mg	0.427	0.504	0.01746	0.010	0.003	0.068
SOD, U/mg	2.26	2.36	0.025	0.007	<0.001	0.041
Adiponectin, mg/g	0.854	1.023	0.0129	<0.001	0.001	0.027
IL-6, pg/mg	8.02	7.63	0.239	0.277	<0.001	0.003
TNF-α, pg/mg	2.47	2.41	0.078	0.632	<0.001	0.883
IL-1β, pg/mg	1.12	1.12	0.091	0.993	0.011	0.861
IL-18, pg/mg	5.45	4.81	0.009	<0.001	<0.001	0.145
NLRP3, pg/mg	6.38	5.74	0.074	0.002	<0.001	0.332
ASC, pg/mg	0.646	0.548	0.0077	<0.001	<0.001	<0.001

^1^*T-AOC* Total antioxidant capacity, *SOD* Superoxide dismutase, *IL* Interleukin, *TNF-α* Tumor necrosis factor α, *NLRP3* NOD-like receptor thermal protein domain associated protein 3, *ASC* Apoptosis associated speck like protein containing a CARD

^2^CON, no supplemental hesperidin; HES, 30 g/d hesperidin. Adipose tissue was biopsied on d 5, 10, and 25 relative to calving (*n* = 16)

^3^Trt, treatment

An interaction (*P* = 0.003) between Trt and time was observed for IL-6 content (Fig. 4A), and it was lower in HES than CON at 5 DIM, whereas IL-6 was not affected by HES at 10 and 25 DIM. TNF-α (Fig. 4B) and IL-1β (Fig. 4C) did not change in response to HES supplementation (*P* > 0.60). Lower concentrations in IL-18 (*P* < 0.001; Fig. 4D), NLRP3 (*P* = 0.002; Fig. 4E), and ASC (*P* < 0.001; Fig. 4F) were detected in cows supplemented with HES. An interaction of Trt × time on ASC concentration was observed. The interaction on ASC resulted in lower ASC in HES cows than control at 5 DIM (*P* < 0.001) and 10 DIM (*P* < 0.01), whereas ASC was not affected by HES at 25 DIM.

### Adipose tissue metabolomic and lipidomic profiles

**Fig. 5:**
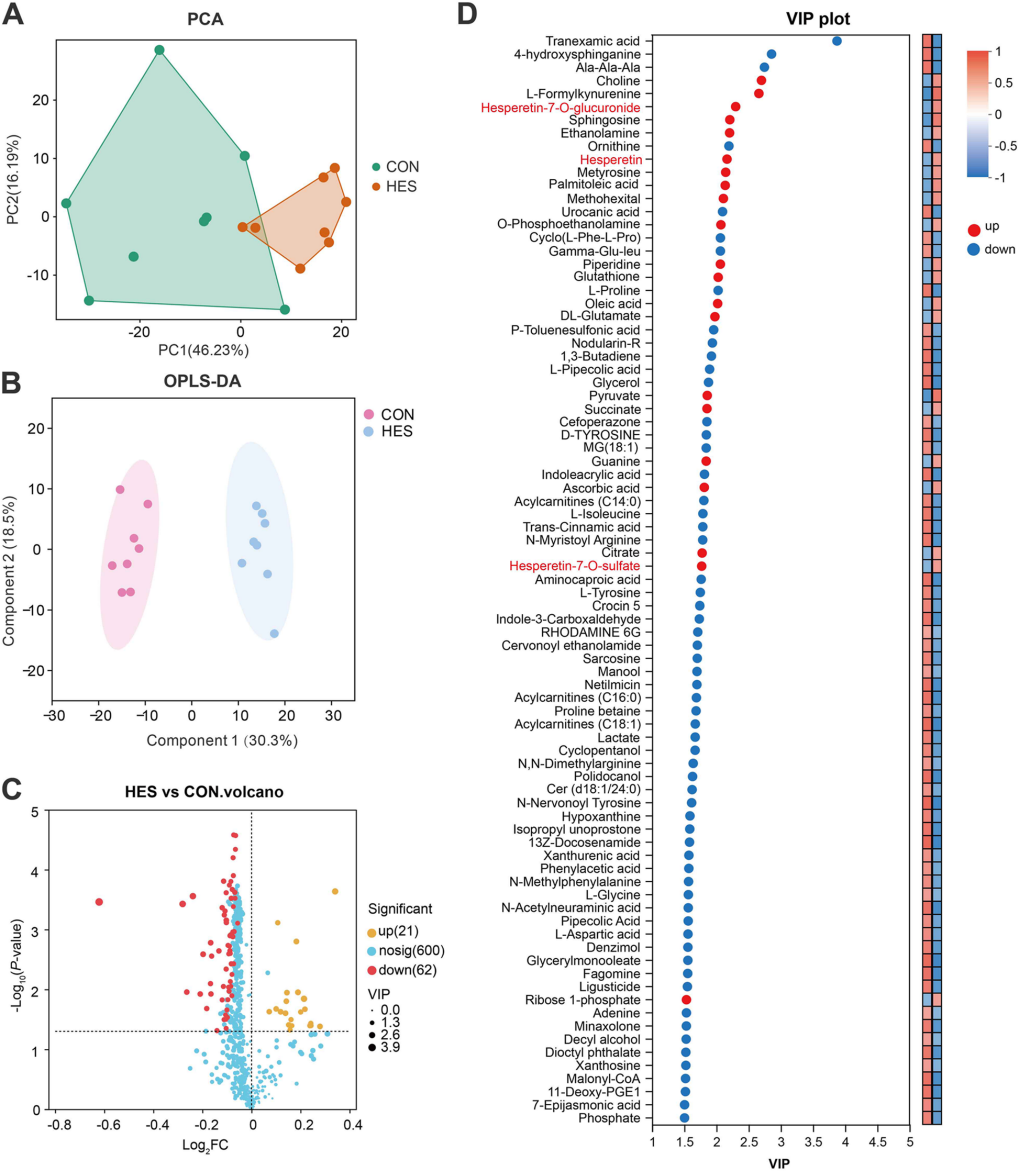
Multivariate and univariate analyses of adipose tissue metabolomic profiles in dairy cows supplemented with hesperidin (HES) or control (CON). **A** Principal component analysis (PCA) score plot of CON and HES groups. **B** Orthogonal partial least squares discriminant analysis (OPLS-DA) score plot of CON and HES groups. **C** Volcano plot of metabolite differences between HES and CON. **D** Variable importance in projection (VIP) plot of the top differential metabolites. Red, metabolites upregulated in HES; blue, metabolites downregulated in HES. All adipose tissue samples for metabolomic analyses were collected at d 10 postpartum

Untargeted metabolomic profiling of adipose tissue initially detected 719 metabolites. After quality control and data preprocessing, a total of 683 reliable metabolites were retained for downstream statistical and pathway analyses. The PCA revealed a clear separation between CON and HES groups, indicating distinct global metabolic profiles (Fig. 5A). This separation was further supported by OPLS-DA, which demonstrated non-overlapping clusters between treatments (Fig. 5B). Differential metabolites were screened (VIP > 1.5 and *P* < 0.05), which identified 21 upregulated and 62 downregulated features in the HES group relative to CON (Fig. 5C and Additional file 4: Table S3).

The volcano plot highlighted a predominant downregulation pattern, consistent with hesperidin-induced suppression of pro-inflammatory and lipotoxic intermediates. Key metabolites driving group separation included sphingolipid-related compounds such as 4-hydroxysphinganine, glycerol-derived intermediates (glycerol, monoacylglycerol 18:1, DG (8:0/0:0/PGF1α)), acylcarnitines (C14:0, C16:0, C18:1), and the ceramide Cer (d18:1/24:0). In addition, several amino acids-related metabolites (ornithine, L-proline, L-isoleucine, L-tyrosine) and organic acids (urocanic acid, xanthurenic acid, hypoxanthine) were decreased, whereas metabolites associated with antioxidative and membrane stability, including glutathione, choline, ethanolamine, O-phosphoethanolamine, succinate, and citrate, were elevated in HES. Notably, fatty acids such as palmitoleic acid and oleic acid also increased, indicating a remodeling of lipid metabolism toward unsaturated species. Additionally, three hesperidin-derived metabolites were detected in adipose tissue, namely hesperetin-7-O-glucuronide, hesperetin, and hesperetin-7-O-sulfate. Compared with the CON group, the relative abundance of all three metabolites was significantly increased in the HES group.

Untargeted lipidomic analysis identified a total of 928 lipid species in adipose tissue. After quality control and data filtering, 808 high-confidence lipids were retained and used for subsequent statistical comparisons and pathway enrichment analyses. These included sphingolipids (SP, *n* = 119), fatty acyls (FA, *n* = 12), glycerophospholipids (GP, *n* = 370), glycerolipids (GL, *n* = 426), and a small number of sterol lipids (ST, *n* = 1) (Additional file 5: Fig. S4A). Among these classes, GP, GL, and ST were the most abundant, collectively accounting for over 98% of the lipidome, whereas fatty acyls, and sterol lipids represented relatively smaller proportions.

**Fig. 6:**
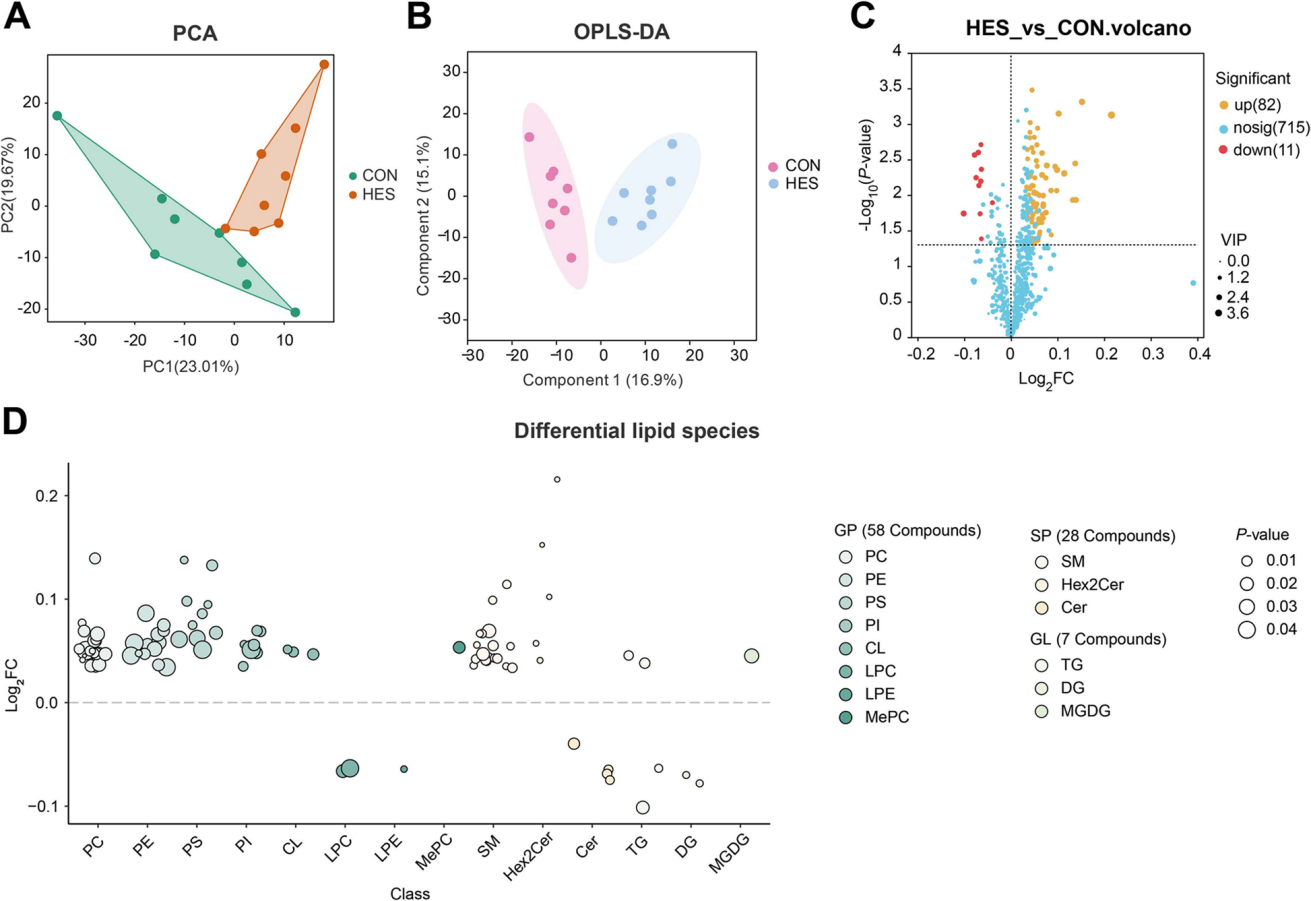
Differential lipid species in adipose tissue after hesperidin supplementation. **A** Principal component analysis (PCA) score plot for CON and HES groups. **B** Orthogonal partial least squares discriminant analysis (OPLS-DA) score plot for CON and HES groups. **C** Volcano plot showing differential lipid species between HES and CON. Red dots: significantly upregulated metabolites; blue dots: significantly downregulated metabolites; orange dots: non-significant metabolites. VIP values are indicated for top differential metabolites. **D** Fold change analysis of differential lipid species across lipid classes. Lipid classes include glycerophospholipids (GP), sphingolipids (SP), glycerolipids (GL). PC, phosphatidylcholine; PE, phosphatidylethanolamine; PS, phosphatidylserine; PI, phosphatidylinositol; CL, cardiolipin; LPC, lysophosphatidylcholine; LPE, lysophosphatidylethanolamine; MePC, methylated phosphatidylcholine; SM, sphingomyelin; Hex2Cer, dihexosylceramide; Cer, ceramide; TG, Triglyceride; DG, diglyceride; MGDG, monogalactosyldiacylglycerol. All adipose tissue samples for lipidomic analyses were collected at d 10 postpartum

Multivariate analysis revealed clear metabolic separation between groups, as shown by PCA (Fig. 6A) and OPLS-DA (Fig. 6B). These analyses demonstrated distinct lipidomic profiles for HES-treated cows compared to controls. Differential lipid species were visualized in a volcano plot (VIP > 1.5 and *P* < 0.05) (Fig. 6C), highlighting 82 upregulated and 11 downregulated lipid species in the HES group (Additional file 6: Table S4). The fold-change analysis of lipid species (Fig. 6D) revealed significant alterations in several lipid classes. Glycerophospholipids, including phosphatidylethanolamines (PE), phosphatidylserines (PS), and phosphatidylinositols (PI), were predominantly upregulated, reflecting enhanced membrane lipid remodeling. Sphingolipids (SM) exhibited strong upregulation, whereas ceramides (Cer) were downregulated, aligning with a reduction in lipotoxic and pro-inflammatory signaling. Glycerolipids such as triglycerides (TG) and diglycerides (DG) showed more complex alterations, with some species (e.g., TG) downregulated, indicating a shift in lipid storage dynamics.

The differential metabolites and lipids identified in adipose tissue are summarized in Table 6. Notable findings include the downregulation of sphingosine, sphinganine, and ceramides (Cer d18:1/16:0, d18:1/18:0), indicating a reduction in ceramide metabolism and sphingolipid signaling. Additionally, glycerol, monoacylglycerol, and acylcarnitines were decreased, which was associated with reduced DG and ceramides, suggesting a shift toward enhanced fatty acid oxidation and mitochondrial lipid remodeling. Choline, ethanolamine, and phosphoethanolamine were upregulated, along with phosphatidylcholine (PC) and PE, reflecting the reinforcement of PUFA-rich phospholipids and plasmalogens. In addition, glutathione and ascorbate were elevated, while lysophospholipids (LPC 18:0, LPC 16:0) and LPE 18:1 were reduced, indicating improved redox status and reduced pro-oxidative lipids. Finally, palmitoleic acid and oleic acid were increased, along with PUFA-TGs, suggesting a shift from saturated to unsaturated lipid species.

**Table 6:** Differential metabolites and lipids in adipose tissue and their overlaps

Metabolomics (differential metabolites)	Lipidomics (differential lipid species)	Overlapping
Sphingosine (↑), Sphinganine (↓)	Cer(d18:1/16:0) (↓), Cer(d18:1/18:0) (↓), Cer(d18:1/24:0) (↓), Cer(m18:1/24:0) (↓)	Reduction in ceramide metabolism and sphingolipid signaling
Glycerol (↓), Monoacylglycerol 18:1 (↓)	DG(19:0/18:1) (↓), DG(18:1/18:3) (↓)	Consistent decrease in DG-related lipotoxic intermediates
Acylcarnitine C14:0 (↓), Acylcarnitine C16:0 (↓), Acylcarnitine C18:1 (↓)	CL(18:2/16:0/20:3/20:3) (↑), CL(20:4/16:0/20:4/24:1) (↑)	Shift toward enhanced fatty-acid oxidation and mitochondrial lipid remodeling
Choline (↑), Ethanolamine (↑), Phosphoethanolamine (↑)	PC(30:0/18:1) (↑), PC(16:2e/19:0) 38:6 (↑), PE(18:0e/18:2) (↑), PE(16:0e/18:2) (↑)	Reinforcement of PUFA-rich phospholipids and plasmalogens
Glutathione (↑), Glutamate (↑), Ascorbate (↑)	LPC(18:0) (↓), LPC(16:0) (↓), LPE(18:1) (↓)	Reduced pro-oxidative lysophospholipids, improved redox status
Citrate (↑), Succinate (↑) (TCA intermediates)	PI(16:0/18:2)(↑), PI(16:0/18:1) (↑), PS(16:0/18:2) (↑), PS(18:2e/18:1) (↑)	Enhanced central carbon metabolism linked to glycerophospholipid remodeling
Palmitoleic acid (↑), Oleic acid (↑)	↑ PUFA-TGs (TG(16:1/14:1/18:3), TG(18:1/18:1/22:4), ↓ SFA-TGs (TG(15:0/8:0/16:0), TG(18:0/18:0/18:0))	Shift from saturated to unsaturated lipid species

### Proteomic profiles of adipose tissue

**Fig. 7:**
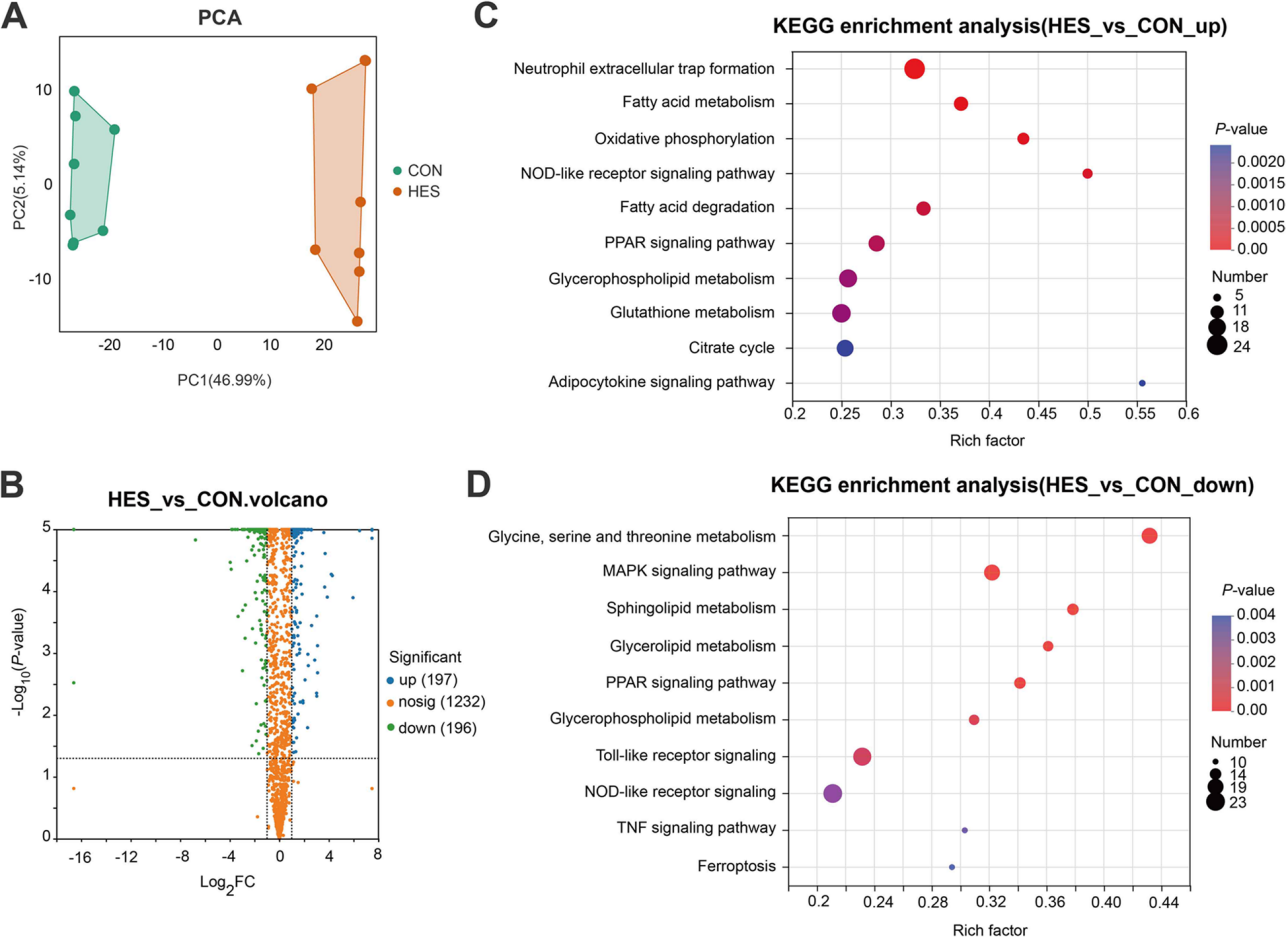
Proteomic analysis of adipose tissue in dairy cows supplemented with hesperidin (HES) or control (CON). **A** Principal component analysis (PCA) score plot for CON and HES groups. **B** Volcano plot of differential proteins between HES and CON. Blue, significantly upregulated proteins (*n* = 197); green, significantly downregulated proteins (*n* = 196); orange, non-significant proteins (*n* = 1232). **C** Top 10 of KEGG pathway enrichment of upregulated proteins, including fatty acid metabolism, oxidative phosphorylation, glycerophospholipid metabolism, glutathione metabolism, citrate cycle, adipocytokine signaling pathway, and related pathways. **D** Top 10 of KEGG pathway enrichment of downregulated proteins, including sphingolipid metabolism, glycerolipid metabolism, glycerophospholipid metabolism, PPAR signaling pathway, Toll-like receptor signaling, NOD-like receptor signaling, TNF signaling pathway, and ferroptosis. All adipose tissue samples for proteomic analyses were collected at d 10 postpartum

Following the exclusion of low-quality spectra, DIA analysis yielded 1,625 distinct proteins. Among these, 1,610 proteins (98.4%) were shared between the CON and HES groups (Additional file 7: Fig. S5A). The majority of identified proteins (90.1%) had molecular weights ranging from 1 to 101 kDa (Additional file 7: Fig. S5B). Principal component analysis further revealed clear separation of protein profiles between CON and HES treatments (Fig. 7A).

In total, 393 differentially expressed proteins (DEPs) were identified from HES to CON, of which 197 were upregulated and 196 were downregulated in the HES group compared with the CON group (Additional file 8: Table S5; Fig. 7B). KEGG enrichment analysis of upregulated proteins indicated significant enrichment in pathways associated with fatty acid metabolism, oxidative phosphorylation, fatty acid degradation, glycerophospholipid metabolism, glutathione metabolism, citrate cycle, and adipocytokine signaling (Fig. 7C). These findings suggest enhanced mitochondrial energy metabolism, phospholipid remodeling, and antioxidant defense in response to hesperidin supplementation. Conversely, downregulated proteins were mainly enriched in sphingolipid metabolism, glycerolipid metabolism, glycerophospholipid metabolism, PPAR signaling pathway, Toll-like receptor signaling, NOD-like receptor signaling, and TNF signaling pathway (Fig. 7D).

**Fig. 8:**
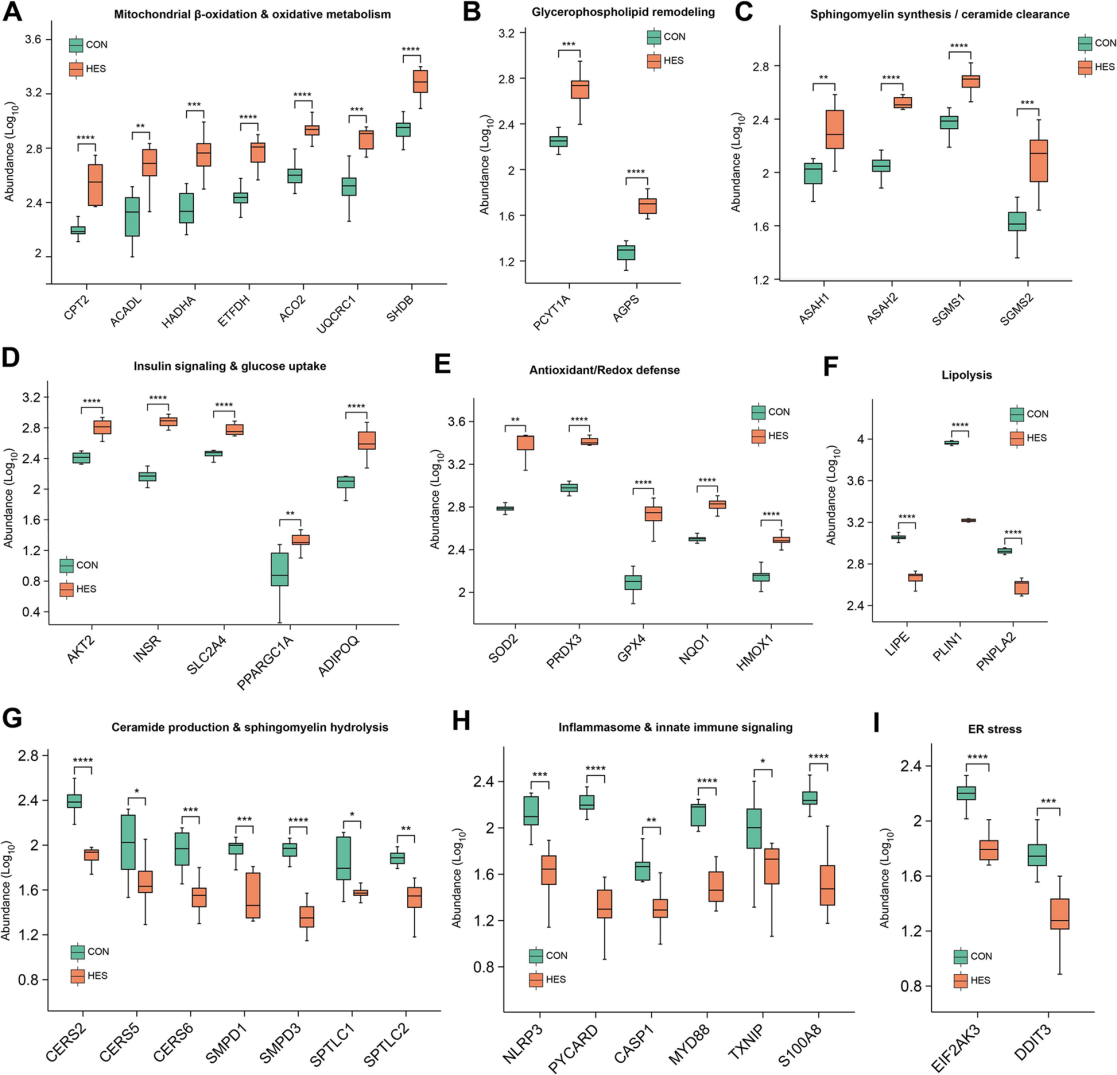
Proteomic analysis of adipose tissue in dairy cows supplemented with hesperidin (HES) or control (CON). Differential protein abundance in adipose tissue of dairy cows supplemented with hesperidin (HES) or control (CON). **A** Mitochondrial β-oxidation and oxidative metabolism proteins, including CPT2, ACADL, HADHA, ETFDH, ACC2, UQCRC1, and SDHB. **B** Glycerophospholipid remodeling proteins, including PCYT1A and AGPS. **C** Sphingomyelin synthesis and ceramide clearance proteins, including ASAH1, ASAH2, SGMS1, and SGMS2. **D** Insulin signaling and glucose uptake proteins, including AKT2, INSR, SLC2A4, PPARGC1A, and ADIPOQ. **E** Antioxidant and redox defense proteins, including SOD2, PRDX3, GPX4, NQO1, and HMOX1. **F** Lipolysis proteins, including LIPE, PLIN1, and PNPLA2. **G** Ceramide production and sphingomyelin hydrolysis proteins, including CERS2, CERS5, CERS6, SMPD1, SMPD3, SPTLC1, and SPTLC2. **H** Inflammasome and innate immune signaling proteins, including NLRP3, PYCARD, CASP1, MYD88, TXNIP, and S100A8. **I** Endoplasmic reticulum stress proteins, including EIF2AK3 and DDIT3. All adipose tissue samples for proteomic analyses were collected at d 10 postpartum

Detailed examination of differential proteins revealed pathway-specific remodeling of adipose tissue in response to hesperidin supplementation (Fig. 8). Proteins involved in mitochondrial β-oxidation and oxidative metabolism [CPT2 (carnitine palmitoyltransferase 2; Log_2_FC= 1.815, *P* < 0.001), ACADL (acyl-CoA dehydrogenase long-chain; Log_2_FC= 1.093, *P* < 0.01), HADHA (hydroxyacyl-CoA dehydrogenase alpha; Log_2_FC= 1.564, *P* < 0.01), ETFDH (electron transfer flavoprotein dehydrogenase; Log_2_FC= 1.635, *P* < 0.001), ACC2 (acetyl-CoA carboxylase 2; Log_2_FC= 1.271, *P* < 0.001), UQCRC1 (ubiquinol-cytochrome C reductase complex III subunit 1; Log_2_FC= 1.03, *P* < 0.001), SDHB (succinate dehydrogenase complex iron sulfur subunit B; Log_2_FC= 1.211, *P* < 0.001)] were markedly upregulated in the HES group (Fig. 8A), consistent with enhanced fatty acid degradation and energy production. Similarly, proteins linked to glycerophospholipid remodeling [PCYT1A (phosphate cytidylyltransferase 1 alpha; Log_2_FC= 1.128, *P* < 0.001), AGPS (alkylglycerol monooxygenase; Log_2_FC= 1.321, *P* < 0.001)] were increased (Fig. 8B), while enzymes promoting sphingomyelin synthesis and ceramide clearance [ASAH1 (N-acylsphingosine amidohydrolase 1; Log_2_FC= 1.105, *P* < 0.01), ASAH2 (N-acylsphingosine amidohydrolase 2; Log_2_FC= 1.447, *P* < 0.001), SGMS1 (sphingomyelin synthase 1; Log_2_FC= 1.386, *P* < 0.001), SGMS2 (sphingomyelin synthase 2; Log_2_FC= 1.376, *P* < 0.001)] were also elevated (Fig. 8C), in agreement with the lipidomic evidence of SM accumulation and reduced ceramide levels.

Key proteins mediating insulin signaling and glucose uptake [AKT2 (AKT serine/threonine kinase 2; Log_2_FC= 1.307, *P* < 0.001), INSR (insulin receptor; Log_2_FC= 1.779, *P* < 0.001), SLC2A4 (solute carrier family 2 member 4; Log_2_FC= 1.57, *P* < 0.001), PPARGC1A (PPARG coactivator 1 alpha; Log_2_FC= 1.15, *P* < 0.01), ADIPOQ (adiponectin, C1Q and collagen domain containing; Log_2_FC= 1.386, *P* < 0.001)] were significantly enriched in HES-treated cows (Fig. 8D), supporting improved insulin sensitivity. In parallel, antioxidant proteins involved in redox defense [SOD2 (superoxide dismutase 2; Log_2_FC= 1.683, *P* < 0.01), PRDX3 (peroxiredoxin 3; Log_2_FC= 1.505, *P* < 0.001), GPX4 (glutathione peroxidase 4; Log_2_FC= 1.476, *P* < 0.001), NQO1 (NAD(P)H quinone dehydrogenase 1; Log_2_FC= 1.524, *P* < 0.001), HMOX1 (heme oxygenase 1; Log_2_FC= 1.286, *P* < 0.001)] showed strong upregulation (Fig. 8E), indicating enhanced oxidative stress protection. Conversely, proteins associated with lipolysis [LIPE (lipase E, hormone sensitive type; Log_2_FC= −1.486, *P* < 0.001), PLIN1 (perilipin 1; Log_2_FC= −1.903, *P* < 0.001), PNPLA2 (patatin like domain 2, triacylglycerol lipase; Log_2_FC= −2.541, *P* < 0.001)] were downregulated (Fig. 8F), consistent with reduced excessive lipid mobilization. Moreover, enzymes involved in ceramide production and sphingomyelin hydrolysis [CERS2 (ceramide synthase 2; Log_2_FC= −1.398, *P* < 0.001), CERS5 (ceramide synthase 5; Log_2_FC= −1.011, *P* < 0.05), CERS6 (ceramide synthase 6; Log_2_FC= −1.224, *P* < 0.001), SMPD1 (sphingomyelin phosphodiesterase 1; Log_2_FC= −1.224, *P* < 0.001), SMPD3 (sphingomyelin phosphodiesterase 3; Log_2_FC= −1.335, *P* < 0.001), SPTLC1 (serine palmitoyltransferase long chain base subunit 1; Log_2_FC= −1.703, *P* < 0.05), SPTLC2 (serine palmitoyltransferase long chain base subunit 2; Log_2_FC= −1.162, *P* < 0.01)] were decreased (Fig. 8G), further confirming the suppression of ceramide-driven lipotoxic signaling. In the inflammatory axis, core proteins of the inflammasome and innate immune signaling pathways [NLRP3 (NLR family pyrin domain containing 3; Log_2_FC= −1.336, *P* < 0.001), PYCARD (PYD and CARD domain containing; Log_2_FC= −1.089, *P* < 0.001), CASP1 (caspase 1; Log_2_FC= −1.481, *P* < 0.01), MYD88 (MYD88 innate immune signal transduction adaptor; Log_2_FC= −1.844, *P* < 0.001), TXNIP (thioredoxin interacting protein; Log_2_FC= −1.045, *P* < 0.05), S100A8 (S100 calcium binding protein A8; Log_2_FC= −1.386, *P* < 0.001)] were downregulated (Fig. 8H), highlighting the anti-inflammatory potential of hesperidin. In addition, proteins associated with endoplasmic reticulum stress [EIF2AK3 (eukaryotic translation initiation factor 2 alpha kinase 3; Log_2_FC= −1.071, *P* < 0.001), DDIT3 (DDIT3 DNA damage inducible transcript 3; Log_2_FC= −1.436, *P* < 0.001)] were also significantly reduced (Fig. 8I), suggesting alleviation of ER stress responses. These results point to a reduction in ceramide-driven lipotoxic signaling, pro-inflammatory pathways, and excessive lipid mobilization.

### Serum lipidomics profiles

Serum lipidomic profiling identified a total of 791 lipid species, among which 699 passed quality filtering for subsequent analysis. These lipids were categorized into five major classes, with GP representing the largest proportion (*n* = 449, 64.23%), followed by SP (*n* = 122, 17.45%), GL (*n* = 107, 15.31%), ST (*n* = 20, 2.86%), and a minor fraction of fatty acyls (*n* = 1, 0.14%) (Additional file 5: Fig. S4B).

**Fig. 9:**
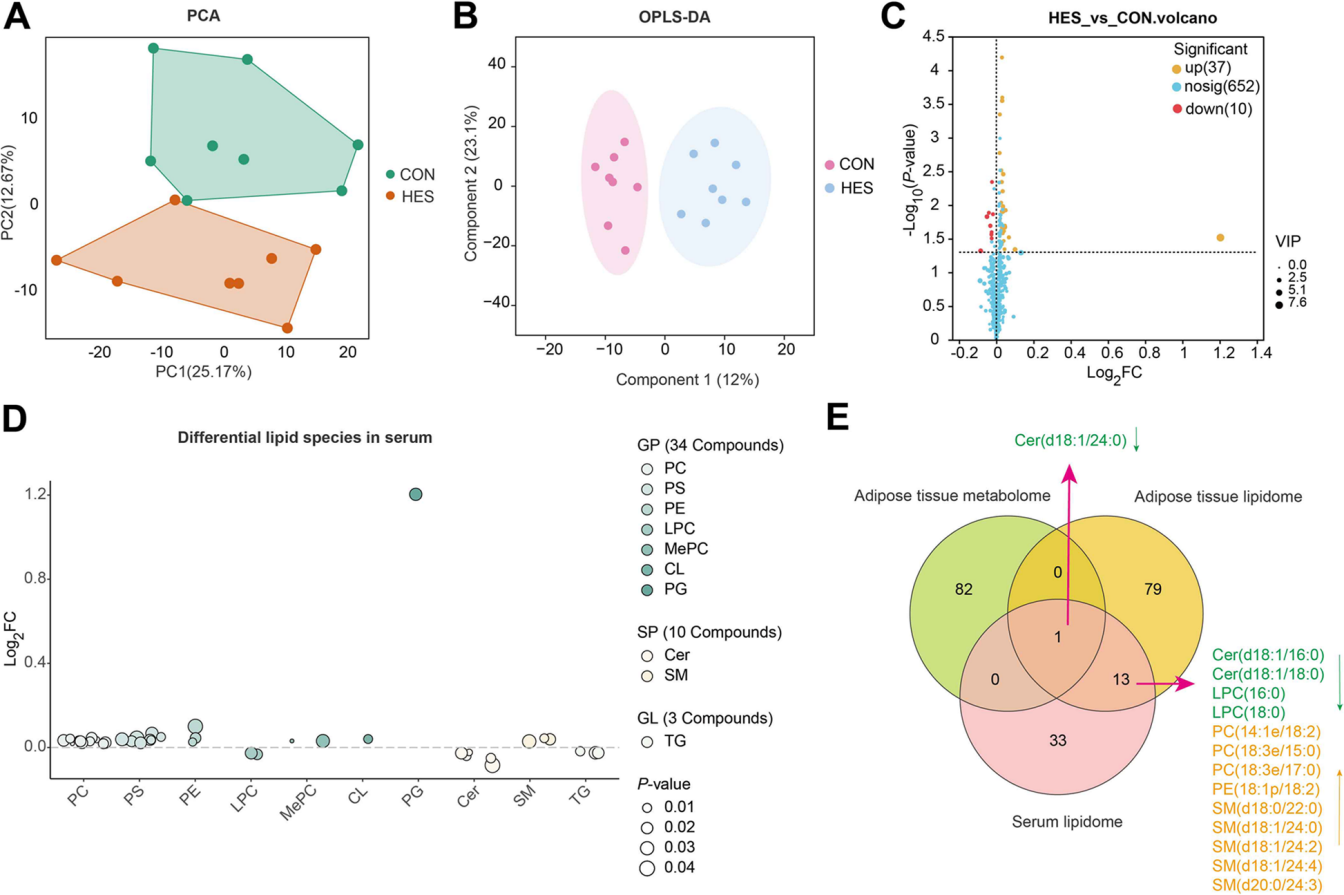
Serum lipidomic profiling and cross-omics integration. **A** Principal component analysis (PCA) score plot showing the separation between CON and HES groups. **B** Orthogonal partial least squares discriminant analysis (OPLS-DA) score plot for CON and HES groups. **C** Volcano plot of differential lipids between HES and CON. Red dots: significantly upregulated lipids (*n* = 37); blue dots: significantly downregulated lipids (*n* = 10); yellow dots: non-significant lipids (*n* = 652). **D** Fold change distribution of differential lipid species across lipid classes, including glycerophospholipids (PC, PS, PE, LPC, MePC, CL, PG), sphingolipids (Cer, SM), and glycerolipids (TG). **E** Venn diagram showing overlapping differential metabolites (metabolome) and lipid species (adipose tissue lipidome, serum lipidome). Shared species include Cer(d18:1/16:0), Cer(d18:1/18:0), Cer(d18:1/24:0), LPC(16:0), LPC(18:0), PC(14:1e/18:2), PC(18:3e/15:0), PC(18:3e/17:0), PE(18:1p/18:2), SM(d18:0/22:0), SM(d18:1/24:0), SM(d18:1/24:2), SM(d18:1/24:4), and SM(d20:0/24:3). All serum samples for lipidomic analyses were collected at d 10 postpartum

Serum lipidomic profiling revealed a clear separation between CON and HES groups, as shown by PCA and OPLS-DA score plots (Fig. 9A and B). The volcano plot highlighted 47 significantly altered lipid species (37 upregulated, 10 downregulated; Fig. 9C), with detailed information provided in Table S6 (Additional file 9). These differential lipids were mainly enriched in glycerophospholipids (e.g., PC, PE, PS, MePC, CL), sphingolipids (SM, Cer), and a small number of glycerolipids (TG) (Fig. 9D). Notably, several sphingomyelin species (e.g., SM(d18:0/22:0), SM(d18:1/24:2), SM(d20:0/24:3)) and phosphatidylcholine species were significantly upregulated, whereas ceramides (Cer(d18:1/16:0), Cer(d18:1/18:0), Cer(d18:1/24:0)) and lysophosphatidylcholines (LPC(16:0), LPC(18:0)) were markedly downregulated. These results suggest a shift in circulating sphingolipid and phospholipid balance in response to hesperidin supplementation.

Integration of metabolomic and lipidomic datasets further demonstrated overlapping differential species across adipose tissue and serum compartments (Fig. 9E). Thirteen lipid molecules were consistently altered in both tissues and circulation, including ceramides, lysophosphatidylcholines, phosphatidylcholines, phosphatidylethanolamines, and sphingomyelins, pointing to coordinated regulation of lipid metabolism.

**Fig. 10:**
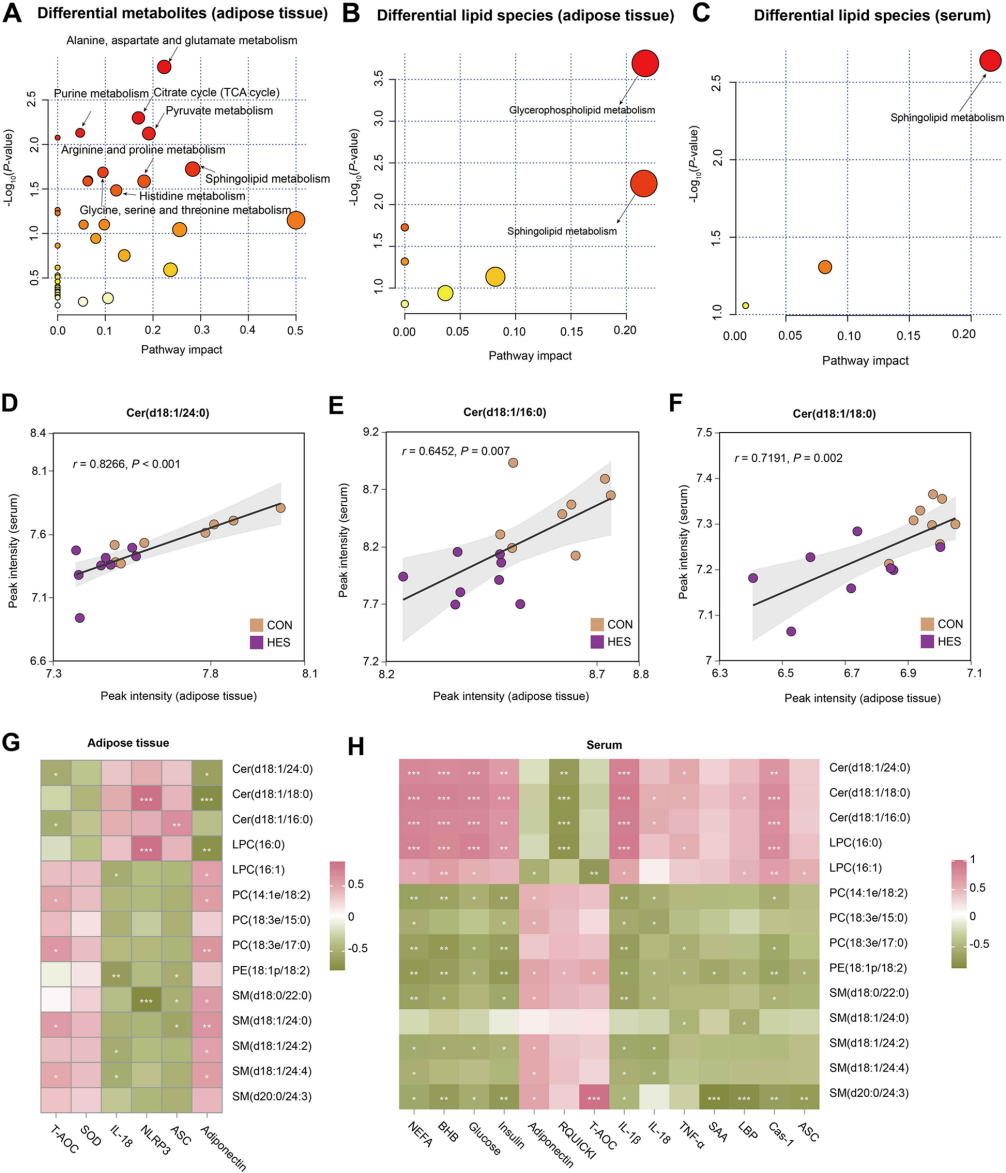
Integrated analysis of differential metabolites and lipids in adipose tissue and serum. **A** Pathway enrichment of differential metabolites in adipose tissue, highlighting TCA cycle, purine metabolism, arginine and proline metabolism, glycine/serine/threonine metabolism, and sphingolipid metabolism. **B** Pathway enrichment of differential lipid species in adipose tissue, with significant enrichment in glycerophospholipid metabolism and sphingolipid metabolism. **C** Pathway enrichment of differential lipid species in serum, with significant enrichment in sphingolipid metabolism. The size of the circles represents the pathway impact, with larger circles indicating pathways that have a greater influence on the overall metabolic processes. The color gradient of the circles reflects the statistical significance of the pathway enrichment (red: highly significant, yellow: less significant). **D**–**F** Correlation between adipose tissue and serum levels of representative ceramide species, including Cer(d18:1/24:0), Cer(d18:1/16:0), and Cer(d18:1/18:0). **G** Heatmap showing correlations between selected differential metabolites/lipids in adipose tissue and indicators of antioxidant status and inflammatory responses. **H** Heatmap showing correlations between selected differential lipid species in serum and metabolic, antioxidant, and inflammatory indicators. ^*^P < 0.05, ^**^P < 0.01, ^***^P < 0.001. All adipose tissue and serum samples for multi-omic analyses were collected at d 10 postpartum

Pathway enrichment analysis further supported these findings. Differential metabolites in adipose tissue were mainly mapped to the TCA cycle, amino acid metabolism, and sphingolipid metabolism (Fig. 10A), while differential lipid species were enriched in glycerophospholipid and sphingolipid metabolism (Fig. 10B). Serum lipid alterations were predominantly associated with sphingolipid metabolism (Fig. 10C). Cross-tissue correlation analysis revealed significant positive associations of ceramides Cer(d18:1/24:0), Cer(d18:1/16:0), and Cer(d18:1/18:0) between adipose tissue and serum (Fig. 10D–F). Moreover, correlation heatmaps demonstrated that altered lipid species in both adipose tissue and serum were strongly linked to antioxidant defense, inflammatory markers, and metabolic indicators (Fig. 10G and H), further indicating that hesperidin-mediated lipid remodeling is closely associated with improved glucose and lipid metabolism, redox and inflammatory status.

### Analysis of hesperidin and its metabolites in serum and adipose tissue

The analysis of hesperidin and its metabolites in serum and adipose tissue on d 10 postpartum revealed significant differences between CON and HES cows (Table 7). Specifically, serum hesperidin levels increased from 6.20 ± 0.292 μg/mL in the CON group to 68.91 ± 3.80 μg/mL in the HES group (*P* < 0.001). Similarly, serum levels of hesperetin, hesperetin-7-O-glucoside, and hesperetin-7-O-sulfate were significantly higher in the HES group (*P* < 0.001 for all comparisons). In adipose tissue, while hesperidin was not detected in either group, significant increases were observed in the metabolites. Hesperetin levels in adipose tissue rose from 0.153 ± 0.0072 μg/mg in the CON group to 3.921 ± 0.3845 μg/mg in the HES group (*P* < 0.001). Both hesperetin-7-O-glucoside and hesperetin-7-O-sulfate also showed significant upregulation in the HES group, with values of 14.705 ± 0.4183 μg/mg (*P* < 0.001) and 17.433 ± 0.6670 μg/mg (*P* < 0.001), respectively, compared to the CON group.

**Table 7:** Analysis of hesperidin and its metabolites in serum and adipose tissue on d 10 postpartum (mean ± SEM)

Item	Treatment^1^		*P*-value
	CON	HES	
Serum, μg/mL			
Hesperidin	6.20 ± 0.292	68.91 ± 3.80	<0.001
Hesperetin	16.1 ± 1.07	175.2 ± 6.36	<0.001
Hesperetin-7-O-glucoside	10.7 ± 1.53	238.2 ± 10.49	<0.001
Hesperetin-7-O-sulfate	11.1 ± 0.26	245.6 ± 9.80	<0.001
Adipose tissue, μg/mg			
Hesperidin	ND^2^	ND^2^	—
Hesperetin	0.153 ± 0.0072	3.921 ± 0.3845	<0.001
Hesperetin-7-O-glucoside	0.846 ± 0.0639	14.705 ± 0.4183	<0.001
Hesperetin-7-O-sulfate	0.590 ± 0.0551	17.433 ± 0.6670	<0.001

^1^CON, no supplemental hesperidin; HES, 30 g/d hesperidin (*n* = 16)

^2^ND, not detected

## Discussion

### HES improved milk composition and systemic metabolic health

In the present study, HES supplementation appears to enhance protein nutrition and energy metabolism in dairy cows, which can explain the observed improvements in milk composition and systemic health. In our study, cows fed HES had higher milk protein concentration and yield alongside lower MUN, suggesting more efficient nitrogen utilization (i.e., less dietary protein wasted as urea). This aligns with reports that citrus peel extracts (rich in polyphenols) can inhibit excessive rumen ammoniagenesis and enhance microbial protein synthesis, thereby increasing milk protein output [17]. Winkler et al. [13] reported that supplementing cows with green tea and curcuma extracts enhanced milk protein yield by 9%. Likewise, Gohlke et al. [28] found that continuous intraduodenal infusion of quercetin elevated milk protein concentration over a 4-week treatment period. Unlike some studies that noted higher milk yield with citrus peel extract supplementation [17, 29], we did not see a significant yield change, possibly due to differences in dosage or lactation stage.

We observed that HES supplementation reduced serum BHB and tended to lower NEFA concentrations, suggesting an attenuation of excessive adipose tissue lipolysis. Similar findings were reported by Ju et al. [17], who showed that citrus peel extract decreased NEFA and BHB in transition cows, and by Ying et al. [18], who observed that citrus extract reduced circulating NEFA in early-lactation cows. Additionally, we observed lower insulin and glucose, and higher adiponectin with HES, resulting in an improved RQUICKI index. This indicates improved insulin sensitivity, consistent with findings for other polyphenols such as quercetin, which has been shown to alleviate insulin resistance and reduce plasma glucose concentrations in late-lactation cows during euglycemic, hyperinsulinemic, and hyperglycemic clamp studies [28].

Hesperidin’s known antioxidant and anti-inflammatory properties likely underlie the marked improvement in systemic health indicators. We found that HES significantly increased T-AOC and reduced proinflammatory cytokines (IL-1β, IL-18, TNF-α, LBP, and SAA), which is in line with the established bioactivity of hesperidin [30, 31]. Importantly, the decrease of IL-1β, IL-18, caspase-1, and ASC observed in the present study suggests that HES may suppress activation of the NLRP3 inflammasome pathway.

### HES alleviated adipose tissue metabolic dysfunction by remodeling lipid metabolism

In the present study, HES supplementation led to marked improvements in adipose tissue metabolic health. Notably, we detected three HES-derived metabolites (hesperetin-7-O-glucuronide, hesperetin, and hesperetin-7-O-sulfate) both in serum and adipose tissue, indicating that dietary HES was bioavailable to this depot. The presence of these metabolites suggests that HES or its bioactive forms directly reach adipose tissue to modulate local metabolism. In line with these findings, our previous work using lactating dairy cows fed CPE also detected hesperetin, a major metabolite of hesperidin, in the circulation [32] and in the milk [33]. This provides additional evidence that dietary flavonoids are absorbed and metabolized in the rumen or intestine [34, 35], and that their bioactive derivatives can be systemically distributed to exert physiological functions.

Consequently, HES-treated cows exhibited enhanced antioxidant capacity (elevated T-AOC and SOD activity) alongside reduced inflammatory activation in adipose tissue. Pro-inflammatory mediators associated with the NLRP3 inflammasome (IL-18, NLRP3, and ASC) were significantly downregulated by HES. Moreover, adiponectin, an insulin-sensitizing and anti-inflammatory adipokine, was upregulated in HES-treated adipose tissue. Given adiponectin’s well-documented metabolic benefits [36, 37], its elevation here signifies an improved anti-inflammatory milieu and insulin-sensitive state in adipose tissue. The proteomic data further corroborated these outcomes: HES increased the expression of antioxidant enzymes (e.g., SOD2, GPX4, and PRDX3) and key insulin signaling proteins (INSR, AKT2, and GLUT4), while repressing inflammasome components (NLRP3, PYCARD) and lipolysis-related proteins (HSL, ATGL). The increase in expression levels of key proteins, as observed in our study, likely involves the modulation of signaling pathways such as the Nrf2 pathway (nuclear factor erythroid 2-related factor 2), which is known to regulate antioxidant gene expression in response to oxidative stress [38]. Hesperidin may activate Nrf2, promoting the transcription of genes involved in antioxidant defense mechanisms, such as *SOD* and *GSH-Px* [39]. This could explain the enhanced antioxidant capacity we observed. Together, these results demonstrate that HES addition effectively ameliorated adipose tissue dysfunction by enhancing redox defenses, promoting an anti-inflammatory phenotype, and improving insulin signaling pathways.

A central finding is that HES caused a profound remodeling of adipose lipid metabolism, most evident in the sphingolipid pathway. Untargeted metabolomics and lipidomics revealed significant decreases in ceramide species (e.g., sphingosine, sphinganine, Cer d18:1/16:0) accompanied by increased SM in the HES group. A similar shift (↓Cer, ↑SM) was observed in serum lipidomic profiles, highlighting the systemic impact of HES. This ceramide-lowering effect is mechanistically important because ceramides are well-established lipotoxic mediators of metabolic dysfunction in rodents and humans [40, 41].

**Fig. 11:**
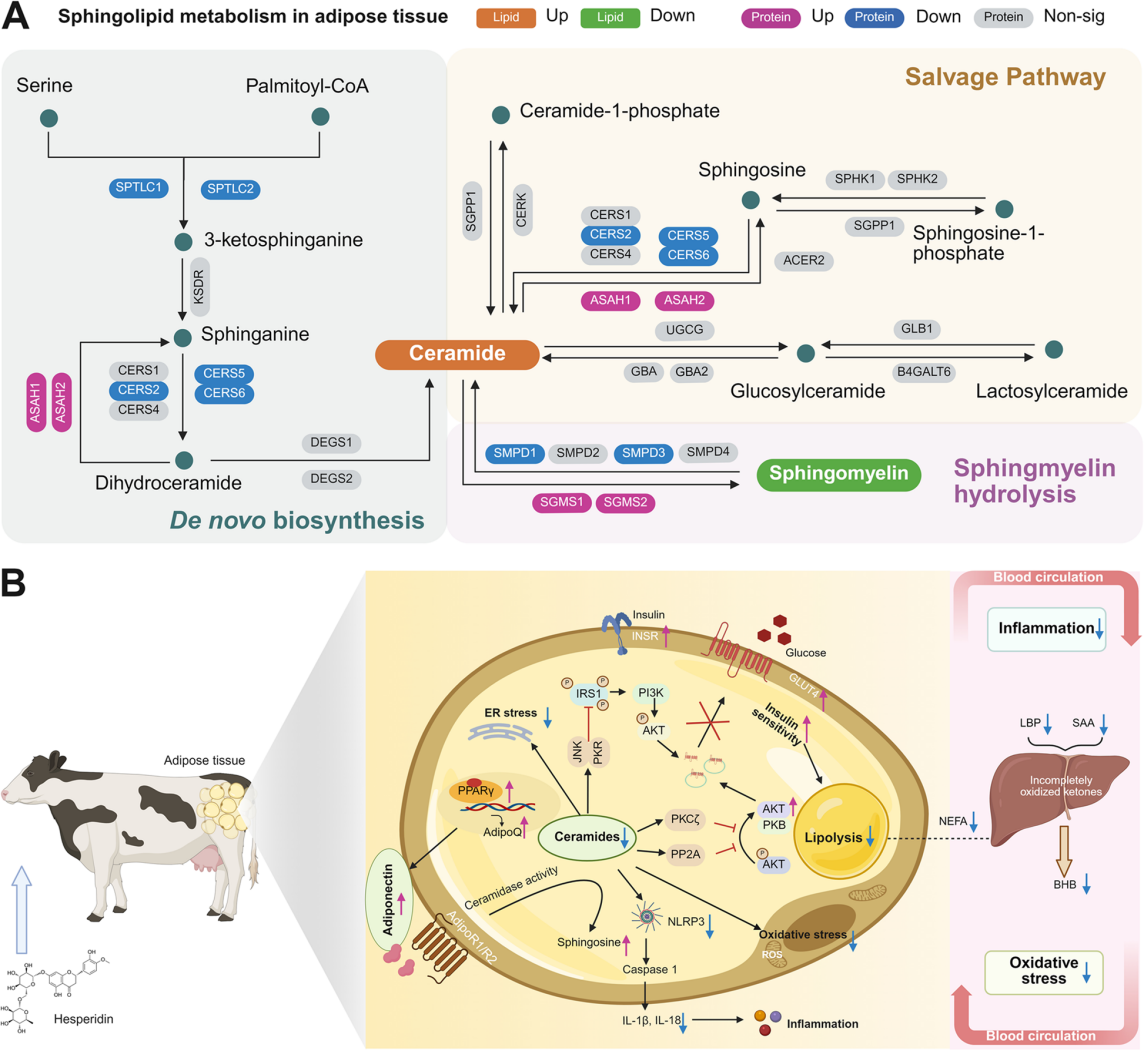
Proposed mechanisms by which hesperidin improves sphingolipid metabolism and metabolic health in adipose tissue of periparturient cows. **A** Integrated lipidomic and proteomic analysis of adipose tissue sphingolipid metabolism. Differential metabolites and proteins are mapped to the de novo biosynthesis, salvage, and hydrolysis pathways. Hesperidin supplementation led to the downregulation of key ceramide synthases (CERS2, CERS5, CERS6) and ceramide accumulation, while promoting sphingomyelin synthesis (SGMS1/2) and hydrolysis (SMPD1/3). These coordinated changes indicate reduced ceramide burden and enhanced sphingolipid remodeling. **B** Proposed working model of hesperidin action in adipose tissue and systemic metabolism. Hesperidin-derived metabolites accumulate in adipose tissue, where they promote PPARγ activation and adiponectin secretion, while attenuating ceramide-driven ER stress, inflammasome activation (NLRP3–caspase-1), oxidative stress, and lipolysis. These changes improve insulin signaling and glucose uptake, lower circulating NEFA and BHBA, and ultimately alleviate systemic oxidative stress and inflammation.

Notably, proteomic analysis indicated that HES upregulated enzymes that dispose of ceramides or divert them into inert storage forms. Acid ceramidase (ASAH1/2) and sphingomyelin synthases (SGMS1/2) were elevated, while simultaneously downregulating key enzymes of ceramide biosynthesis (SPTLC1/2, CERS2/5/6) and sphingomyelinases (Fig. 11A). This coordinated enzyme regulation explains the observed accumulation of sphingomyelins at the expense of ceramides in HES-treated adipose. By converting and degrading ceramides, HES effectively neutralized a major source of lipotoxic stress in adipose tissue. Beyond protein expression changes, two additional mechanisms may further contribute to ceramide reduction. First, HES tended to decrease circulating NEFA, the main lipid precursors for ceramide synthesis [42]; thus, reduced substrate availability may limit ceramide accumulation. Second, the marked increase in adiponectin observed here is relevant because adiponectin receptors (AdipoR1/2) possess intrinsic ceramidase activity [43, 44], which promotes the hydrolysis of ceramides into sphingosine and fatty acids (Fig. 11B). Clinical studies have found that adiponectin levels are inversely correlated with ceramide accumulation in obese, insulin-resistant individuals [45–47]. Conversely, adipose ceramide overload is strongly associated with insulin resistance and type 2 diabetes in humans [48]. Our recent longitudinal study in postpartum dairy cows demonstrated that circulating adiponectin levels were inversely correlated with serum ceramide species, particularly Cer(d20:0/18:0), Cer(d18:1/16:0), and Cer(d18:0/24:0) [8].

Excess ceramide in adipose tissue can activate inflammatory pathways and impair insulin signaling; in particular, ceramide accumulation has been shown to trigger the NLRP3 inflammasome and inhibit Akt/PKB, leading to inflammation-induced insulin resistance [49]. In our study, HES-induced ceramide depletion aligns with the suppression of inflammasome markers and improved insulin signaling noted above. Thus, the HES-driven shift away from ceramides toward sphingomyelins likely underlies a significant part of its metabolic benefits, by relieving ceramide-induced inhibition of insulin signaling and inflammation.

Ceramides concurrently provoke oxidative stress in adipose tissue by disrupting mitochondrial function. Studies show that ceramide is a key driver of intracellular ROS generation, largely by impairing the mitochondrial electron transport chain and increasing outer mitochondrial membrane permeability [50–52]. In adipocytes, even short-chain ceramide analogs acutely inhibit components of the electron transport chain and β-oxidation, undermining respiratory capacity [53]. This ceramide-induced mitochondrial dysfunction leads to excessive ROS leakage and diminished ATP production, creating an oxidative stress state. Importantly, a study in dairy cows highlight those disruptions in adipose ceramide metabolism coincide with heightened oxidative stress. In cows with negative energy balance or subclinical ketosis, adipose tissue ceramide and sphingolipid accumulation correlates with increased markers of oxidative stress and inflammation, along with reduced insulin sensitivity [9]. The HES-treated cows in our study exhibited increased antioxidant enzyme activities (SOD, T-AOC), indicating enhanced capacity to counter ROS. The reduction of ceramides (and upregulation of enzymes like ASAH1/2 and SGMS1/2) likely contributed to lowering oxidative stress, which in turn reduces further ceramide synthesis and inflammasome activation.

Ceramides are known to activate the unfolded protein response (UPR) via key sensors such as PERK and CHOP, and prolonged ER stress contributes to apoptosis, metabolic dysregulation, and inflammation in many mammalian tissues [54, 55]. In our study, HES supplementation lowered adipose tissue markers of ER stress (PERK and CHOP) in parallel with decreased ceramide levels, suggesting that HES helps restore ER homeostasis. Moreover, lower lipolysis (as evidenced by reduced NEFA) may reduce the flux of saturated fatty acid precursors that feed into the ceramide biosynthesis pathway, thereby lessening ER burden.

Hesperidin appears to remodel adipose tissue glycerophospholipid composition while dampening lipotoxic signaling in fat depots. Similarly, in dairy cows supplemented with naringin, adipose tissue levels of PC and PE increased significantly even as TG content declined, indicating a shift toward greater membrane glycerophospholipid content [20]. Notably, enhancing PC/PE availability in adipose membranes may alleviate obesity-related endoplasmic reticulum stress and improve insulin sensitivity in adipocytes [56]. Glycerophospholipids participate in signaling cascades that regulate immune activity [57]. Thus, elevated PE and PC concentrations may influence adipose immune signaling, supporting immune balance, and attenuating inflammation. By maintaining membrane stability and phospholipid integrity, hesperidin may enhance the resistance of adipose tissue to oxidative and inflammatory stress.

Taken together, these findings indicate that hesperidin supplementation acts on multiple levels of adipose and systemic metabolism, including modulation of lipolysis, enhancement of antioxidant and anti-inflammatory defenses, remodeling of sphingolipid and glycerophospholipid pathways, and reinforcement of insulin sensitivity. Importantly, these tissue-level adaptations were reflected in systemic improvements, including lower BHB, enhanced adiponectin, and improved milk protein yield. By linking local adipose remodeling with whole-body metabolic outcomes, our results highlight hesperidin as a promising nutritional strategy to buffer the metabolic challenges of the periparturient period in dairy cows, and they open new avenues for exploring phytochemicals as modulators of lipid and sphingolipid metabolism in ruminants. These findings also open new avenues for exploring phytochemicals as modulators of lipid and sphingolipid metabolism in ruminants, with a broader scope for improving long-term animal health and productivity. However, further research is needed to explore the long-term effects of hesperidin supplementation on sustained metabolic health, lactation performance, and immune function beyond the immediate postpartum period. Such long-term studies could provide valuable insights into the potential lasting benefits of hesperidin and its role in managing chronic metabolic challenges in dairy cows. Additionally, the precise molecular and cellular mechanisms by which hesperidin exerts these effects, particularly in regulating oxidative-antioxidative balance and lipid metabolism, remain to be fully understood. Further studies at the cellular level are essential to elucidate the signaling pathways and enzymatic regulation involved, providing a more detailed understanding of how hesperidin impacts metabolic health at the molecular scale.

## Conclusions

Hesperidin supplementation in periparturient dairy cows improved milk protein output and metabolic health by enhancing antioxidant defenses, reducing inflammation, and reprogramming adipose tissue metabolism. Multi-omics analyses revealed lipid metabolism remodeling, including shifts towards sphingomyelins and glycerophospholipids, and upregulation of mitochondrial and fatty acid oxidation pathways. These findings suggest that hesperidin supplementation can optimize metabolic homeostasis and enhance overall cow health during the transition period, offering a potential nutritional strategy for improving dairy cow productivity. The results highlight hesperidin as a promising intervention to address metabolic challenges in dairy cows, potentially offering long-term benefits for farm management. Future research should explore how hesperidin interacts with liver metabolism and systemic metabolic adaptation to further enhance dairy production efficiency and animal welfare.

## Supplementary Information

Additional file 1: Table S1. Experimental diets (% of DM unless otherwise indicated) fed to cows during the dry and lactation period.

Additional file 2: Table S2. Detailed parameters for ELISA kit validation and experimental design in the analysis of adiponectin, insulin, and inflammatory markers.

Additional file 3: Fig. S1. Effects of dietary supplementation with hesperidin on serum antioxidant status in periparturient cows. Fig. S2. Effects of dietary supplementation with hesperidin on serum NLRP3 inflammasome and proinflammatory cytokines in periparturient cows. Fig. S3. Effects of dietary supplementation with hesperidin on serum acute phase protein in periparturient cows.

Additional file 4: Table S3. Differential metabolites in adipose tissue samples between CON and HES cows.

Additional file 5: Fig. S4. Lipid classification in adipose tissue and serum.

Additional file 6: Table S4. Differential lipid species in adipose tissue samples between CON and HES cows.

Additional file 7: Fig. S5. Proteomic overview of adipose tissue in control (CON) and hesperidin (HES) cows.

Additional file 8: Table S5. Differentially expressed proteins in adipose tissue of dairy cows.

Additional file 9: Table S6. Differential lipid species in serum samples between CON and HES cows.

## Data Availability

The datasets used and/or analyzed during the current study are available from the corresponding author on reasonable request.

## Abbreviations

ACC2: Acetyl-CoA carboxylase 2
ACADL: Acyl-CoA dehydrogenase long chain
ASC: Apoptosis-associated speck-like protein containing a CARD
BCS: Body condition score
BHB: β-hydroxybutyrate
Cas-1: Caspase-1
Cer: Ceramide
CON: Control group
DIM: Days in milk
DMI: Dry matter intake
ECM: Energy-corrected milk
ELISA: Enzyme-linked immunosorbent assay
ETFDH: Electron transfer flavoprotein dehydrogenase
FCM: Fat-corrected milk
GSH-Px: Glutathione peroxidase
GPX4: Glutathione peroxidase 4
HADHA: Hydroxyacyl-CoA dehydrogenase trifunctional multienzyme complex subunit alpha
HES: Hesperidin
IL: Interleukin
INSR: Insulin receptor
LC–MS/MS: Liquid chromatography–tandem mass spectrometry
LPC: Lysophosphatidylcholine
LPE: Lysophosphatidylethanolamine
LBP: Lipopolysaccharide-binding protein
MUN: Milk urea nitrogen
NEB: Negative energy balance
NEFA: Non-esterified fatty acids
NLRP3: NOD-like receptor family pyrin domain-containing protein 3
OPLS-DA: Orthogonal partial least squares-discriminant analysis
PCA: Principal component analysis
PC: Phosphatidylcholine
PE: Phosphatidylethanolamine
PI: Phosphatidylinositol
PRDX3: Peroxiredoxin-3
PS: Phosphatidylserine
PUFA: Polyunsaturated fatty acids
RQUICKI: Revised quantitative insulin sensitivity check index
ROS: Reactive oxygen species
SAA: Serum amyloid A
SCC: Somatic cell count
SDHB: Succinate dehydrogenase complex subunit B
SM: Sphingomyelin
SOD: Superoxide dismutase
SOD2: Superoxide dismutase 2
SP: Sphingolipids
T-AOC: Total antioxidant capacity
TC: Total cholesterol
TG: Triglyceride
TMR: Total mixed ration
TNF-α: Tumor necrosis factor alpha
UHPLC: Ultra-high-performance liquid chromatography
VIP: Variable importance in projection
