# Supplementary material for: Hesperidin alleviates systemic inflammation and oxidative stress by remodeling adipose tissue lipid metabolism in periparturient dairy cows
Source: J Anim Sci Biotechnol. 2026 Apr 5;17:58. doi: 10.1186/s40104-026-01372-4 (PMC13050489; doi:10.1186/s40104-026-01372-4)
Supplement: Supplementary file 1 — Additional file 1: Table S1. Experimental diets (% of DM unless otherwise indicated) fed to cows during the dry and lactation period. [file 40104_2026_1372_MOESM1_ESM.docx]

Table S1. Experimental diets (% of DM unless otherwise indicated) fed to cows during the dry and lactation period.

| Item^1^ | Prepartum | Early lactation |
| --- | --- | --- |
| Ingredient, % of DM | |  |
| Corn silage | 25.7 | 30.4 |
| Alfalfa silage | - | 7.84 |
| Oat Hay | 45.6 | - |
| Alfalfa hay | - | 10.4 |
| Soybean meal | 7.68 | 16.2 |
| Soybean hull | 7.74 | 3.65 |
| Crushed corn | 3.88 | 9.46 |
| Corn steam flakes | - | 9.30 |
| Whole cottonseed | - | 5.36 |
| Corn gluten meal | 4.08 | - |
| Sugarcane molasses | - | 2.59 |
| DCAD supplement | 1.01 | 1.01 |
| Mineral and vitamin mix^2^ | 4.31 | 3.70 |
| Chemical composition, % of DM |  |  |
| DM, as fed % | 51.2 | 47.8 |
| CP | 15.6 | 17.5 |
| NFC^3^ | 33.2 | 38.5 |
| NDF | 43.5 | 33.2 |
| ADF | 23.5 | 19.3 |
| EE | 3.48 | 5.10 |
| Ash | 5.10 | 5.68 |
| NE_L_ (Mcal/kg of DM) | 1.38 | 1.69 |
| DCAD^4^ | −98.1 | 291 |

^1^DM = dry matter; DCAD = dietary cation-anion difference; CP = crude protein; NFC = non-fiber carbohydrates; NDF = neutral detergent fiber; ADF = acid detergent fiber; EE = ether extract; NE_L_ = net energy for lactation.

^2^Prepartum premix: 1 kg of premix included 1,400,000 IU Vitamin A, 437,500 IU Vitamin D, 19,000 IU Vitamin E, 2,000 mg Cu, 3,600 mg Mn, 9,000 mg Zn, 120 mg Se, 160 mg I, 100 mg Co. Early lactation premix: 1 kg of premix included 150,000 IU Vitamin A, 35,000 IU Vitamin D, 2,000 IU Vitamin E, 250 mg Cu, 500 mg Mn, 1,000 mg Zn, 20 mg Se, 40 mg I, 25 mg Co.

^3^NFC = DM − (ash + CP + EE + NDF).

^4^DCAD (mEq/kg of DM) = (Na + K) − (Cl + S), where the unit of Na, K, Cl, and S is mEq/kg of DM.
