# Supplementary material for: Hesperidin alleviates systemic inflammation and oxidative stress by remodeling adipose tissue lipid metabolism in periparturient dairy cows
Source: J Anim Sci Biotechnol. 2026 Apr 5;17:58. doi: 10.1186/s40104-026-01372-4 (PMC13050489; doi:10.1186/s40104-026-01372-4)
Supplement: Supplementary file 2 — Additional file 2: Table S2. Detailed parameters for ELISA kit validation and experimental design in the analysis of adiponectin, insulin, and inflammatory markers. [file 40104_2026_1372_MOESM2_ESM.docx]

Table S2. Detailed parameters for ELISA kit validation and experimental design in the analysis of adiponectin, insulin, and inflammatory markers.

| Item | Adiponectin | Insulin | IL-1β | IL-2 | IL-6 | IL-18 | TNF-α | SAA | LBP | Cas-1 | ASC | NLRP3 |
| --- | --- | --- | --- | --- | --- | --- | --- | --- | --- | --- | --- | --- |
| Standard Curve Range | 1-100 mg/mL; 1-100 mg/g | 1-100μU/mL | 1-1000 pg/mL； 1-1000 pg/mg | 1-500 pg/mL | 1-500 pg/mL | 2-1000 pg/mL; 2-1000 pg/mg; | 1-500 pg/mL; 1-500 pg/mg; | 1-200 ng/mL | 2-1000 ng/mL | 1-500 pg/mL | 0.5-1000 pg/mL; 0.5-1000 pg/mg | 1-200 pg/mg |
| Validation | Verified by Manufacturer | Verified by Manufacturer | Internal validation, reference method available | Verified by Manufacturer | Internal validation, cross-referenced | Internal validation, cross-referenced | Verified by Manufacturer | Internal validation, cross-referenced | Verified by Manufacturer | Verified by Manufacturer | Verified by Manufacturer | Verified by Manufacturer |
| Number of Replicates | 3 | 3 | 3 | 3 | 3 | 3 | 3 | 3 | 3 | 3 | 3 | 3 |
| Single Sample Run on All Plates | Yes | Yes | Yes | Yes | Yes | Yes | Yes | Yes | Yes | Yes | Yes | Yes |
| Plate Corrections | Yes | Yes | Yes | Yes | Yes | Yes | Yes | Yes | Yes | Yes | Yes | Yes |
| Positive Control | Yes | Yes | Yes | Yes | Yes | Yes | Yes | Yes | Yes | Yes | Yes | Yes |
| Negative Control | Yes | Yes | Yes | Yes | Yes | Yes | Yes | Yes | Yes | Yes | Yes | Yes |
