# Supplementary material for: Hesperidin alleviates systemic inflammation and oxidative stress by remodeling adipose tissue lipid metabolism in periparturient dairy cows
Source: J Anim Sci Biotechnol. 2026 Apr 5;17:58. doi: 10.1186/s40104-026-01372-4 (PMC13050489; doi:10.1186/s40104-026-01372-4)
Supplement: Supplementary file 3 — Additional file 3: Fig. S1. Effects of dietary supplementation with hesperidin on serum antioxidant status in periparturient cows. Fig. S2. Effects of dietary supplementation with hesperidin on serum NLRP3 inflammasome and proinflammatory cytokines in periparturient cows. Fig. S3. Effects of dietary supplementation with hesperidin on serum acute phase protein in periparturient cows. [file 40104_2026_1372_MOESM3_ESM.docx]

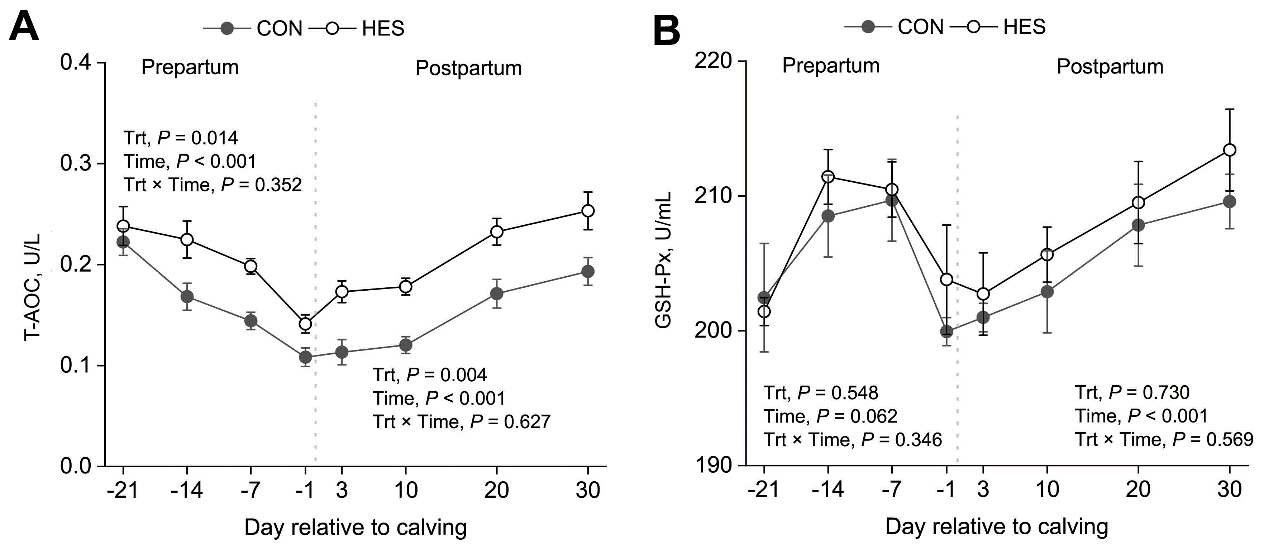


**Fig. S1.** Effects of dietary supplementation with hesperidin on serum antioxidant status in periparturient cows. (A) T-AOC, (B) GSH-Px. Cows (*n* = 32) were assigned to 1 of 2 dietary treatments (TMR with or without HES) in a randomized complete block design. Serum indices were measured on d −21, −14, −7, −1, 3, 10, 20, and 30 relative to calving. CON, no supplemental hesperidin; HES, 30 g/d hesperidin; Trt, treatment; T-AOC, total antioxidant capacity; GSH-Px, glutathione peroxidase. Error bars represent SE of the LSM.


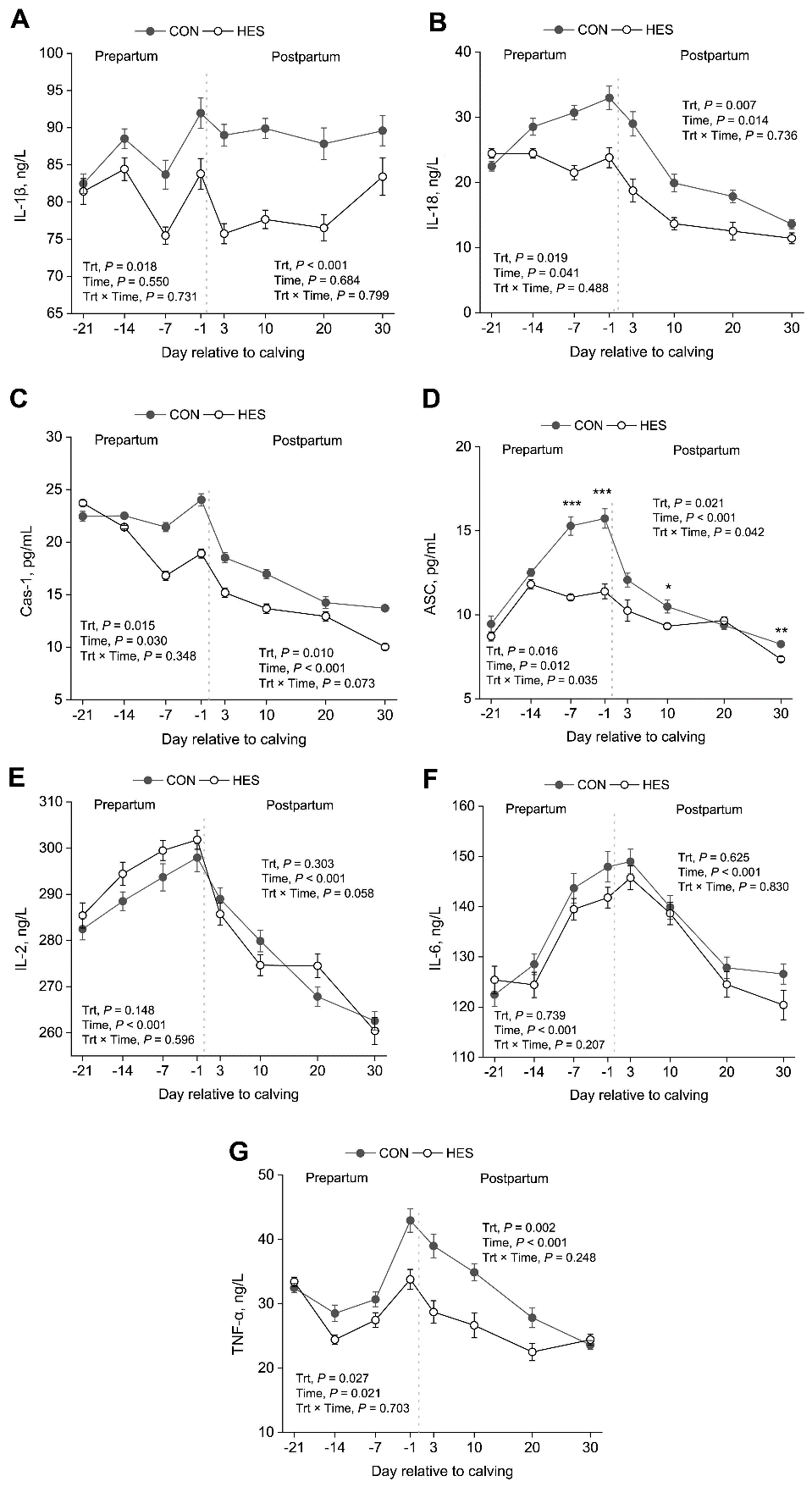


**Fig. S2.** Effects of dietary supplementation with hesperidin on serum NLRP3 inflammasome and proinflammatory cytokines in periparturient cows. (A) IL-1β, (B) IL-18, (C) Cas-1, (D) ASC, (E) IL-2, (F) IL-6, (G) TNF-α. Cows (*n* = 32) were assigned to 1 of 2 dietary treatments (TMR with or without HES) in a randomized complete block design. Serum indices were measured on d −21, −14, −7, −1, 3, 10, 20, and 30 relative to calving. CON, no supplemental hesperidin; HES, 30 g/d hesperidin; Trt, treatment; IL, interleukin, TNF-α, tumor necrosis factor α; Cas-1, Caspase-1; ASC, apoptosis-associated speck-like protein containing a CARD. Error bars represent SE of the LSM. If interactions between treatment and time were significant (*P* < 0.05), data were analyzed individually for each time point using t-test (**P* < 0.05; **P* < 0.01; ****P*<0.001).


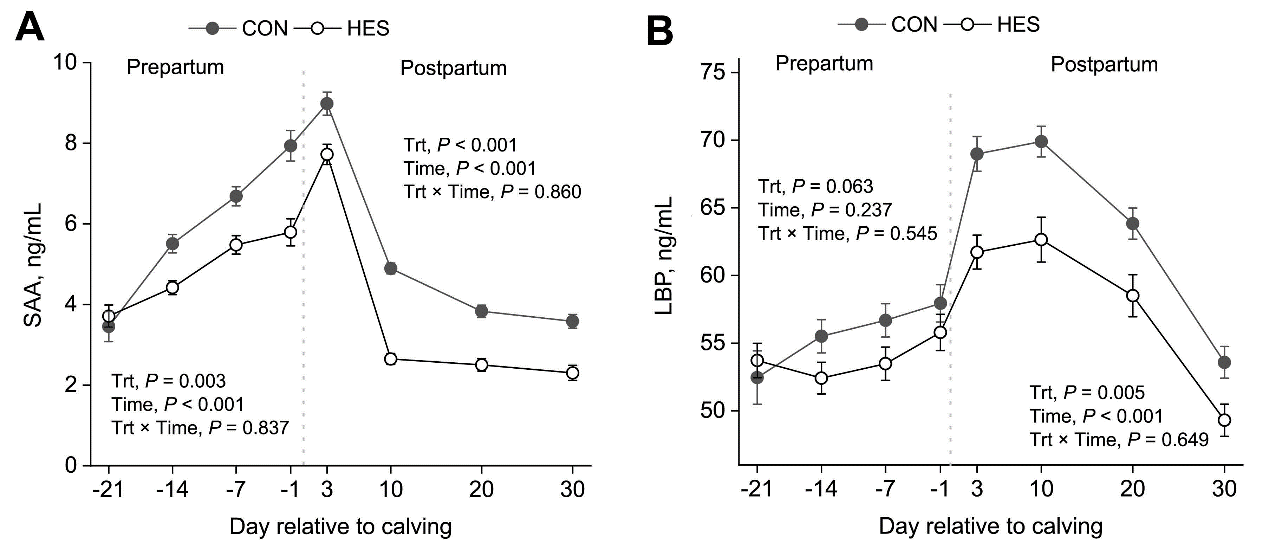


**Fig. S3.** Effects of dietary supplementation with hesperidin on serum acute phase protein in periparturient cows. (A) SAA, (B) LBP. Cows (*n* = 32) were assigned to 1 of 2 dietary treatments (TMR with or without HES) in a randomized complete block design. Serum indices were measured on d −21, −14, −7, −1, 3, 10, 20, and 30 relative to calving. CON, no supplemental hesperidin; HES, 30 g/d hesperidin; Trt, treatment; SAA, serum amyloid A; LBP, lipopolysaccharide binding protein. Error bars represent SE of the LSM.
