# Supplementary material for: Hesperidin alleviates systemic inflammation and oxidative stress by remodeling adipose tissue lipid metabolism in periparturient dairy cows
Source: J Anim Sci Biotechnol. 2026 Apr 5;17:58. doi: 10.1186/s40104-026-01372-4 (PMC13050489; doi:10.1186/s40104-026-01372-4)
Supplement: Supplementary file 4 — Additional file 4: Table S3. Differential metabolites in adipose tissue samples between CON and HES cows. [file 40104_2026_1372_MOESM4_ESM.docx]

Table S3. Differential metabolites in adipose tissue samples between CON and HES cows.

| Metabolites | VIP | *P*-value | Log2(FC) | Regulate |
| --- | --- | --- | --- | --- |
| Tranexamic acid | 3.8739 | 0.000346 | -0.621 | down |
| 4-hydroxysphinganine | 2.8567 | 0.000374 | -0.2807 | down |
| Ala-Ala-Ala | 2.7461 | 0.000277 | -0.2389 | down |
| Choline | 2.6999 | 0.01432 | 0.2139 | up |
| L-Formylkynurenine | 2.66 | 0.000231 | 0.3408 | up |
| Hesperetin-7-O-glucuronide | 2.2991 | 0.01117 | 0.1893 | up |
| Sphingosine | 2.2082 | 0.00158 | 0.1828 | up |
| Ethanolamine | 2.205 | 0.04148 | 0.279 | up |
| Ornithine | 2.1945 | 0.002583 | -0.1973 | down |
| Hesperetin | 2.1648 | 0.02359 | 0.1186 | up |
| Metyrosine | 2.1443 | 0.02493 | 0.1386 | up |
| Matrine | 2.1366 | 0.0113 | 0.1444 | up |
| Palmitoleic acid | 2.1085 | 0.01573 | 0.1419 | up |
| Urocanic acid | 2.0954 | 0.001659 | -0.1665 | down |
| O-Phosphoethanolamine | 2.0698 | 0.03906 | 0.1517 | up |
| Cyclo(L-Phe-L-Pro) | 2.0625 | 0.01106 | -0.2638 | down |
| Gamma-Glu-leu | 2.0621 | 0.01192 | -0.2091 | down |
| Piperidine | 2.0617 | 0.02113 | 0.1002 | up |
| Glutathione | 2.0278 | 0.02139 | 0.2176 | up |
| L-Proline | 2.0269 | 0.000699 | -0.1027 | down |
| Oleic acid | 2.0195 | 0.02349 | 0.0722 | up |
| DL-Glutamate | 1.9771 | 0.04024 | 0.238 | up |
| P-Toluenesulfonic Acid | 1.9578 | 0.0119 | -0.1654 | down |
| Nodularin-R | 1.9376 | 0.002276 | -0.1328 | down |
| 1,3-Butadiene | 1.9233 | 0.000432 | -0.1195 | down |
| L-Pipecolic acid | 1.8948 | 0.001265 | -0.0873 | down |
| Glycerol | 1.8775 | 0.000156 | -0.1137 | down |
| Pyruvate | 1.8578 | 0.00077 | 0.1069 | up |
| Succinate | 1.8538 | 0.03683 | 0.2417 | up |
| Cefoperazone | 1.8531 | 0.02083 | -0.1826 | down |
| D-TYROSINE | 1.8441 | 0.000764 | -0.1025 | down |
| MG(18:1) | 1.8416 | 0.007991 | -0.1672 | down |
| Guanine | 1.8413 | 0.04013 | 0.2424 | up |
| Indoleacrylic acid | 1.8144 | 0.000301 | -0.0826 | down |
| Ascorbic acid | 1.8118 | 0.02378 | 0.2003 | up |
| Acylcarnitines (C14:0) | 1.805 | 0.002774 | -0.1626 | down |
| L-Isoleucine | 1.79 | 0.001082 | -0.0711 | down |
| Trans-Cinnamic acid | 1.7883 | 0.001314 | -0.0761 | down |
| N-Myristoyl Arginine | 1.7869 | 0.000572 | -0.1108 | down |
| Citrate | 1.778 | 0.04748 | 0.1571 | up |
| Hesperetin-7-O-sulfate | 1.7713 | 0.03188 | 0.161 | up |
| Aminocaproic acid | 1.7635 | 0.000239 | -0.0678 | down |
| L-Tyrosine | 1.7497 | 0.003735 | -0.0825 | down |
| Crocin 5 | 1.7416 | 0.004568 | -0.1033 | down |
| Indole-3-Carboxaldehyde | 1.7344 | 0.000489 | -0.108 | down |
| RHODAMINE 6G | 1.7105 | 0.04871 | -0.1407 | down |
| Cervonoyl ethanolamide | 1.7043 | 0.0107 | -0.0915 | down |
| Sarcosine | 1.7027 | 0.002259 | -0.088 | down |
| Manool | 1.6985 | 0.02933 | -0.0987 | down |
| Netilmicin | 1.6975 | 0.000214 | -0.0787 | down |
| Acylcarnitines (C16:0) | 1.6894 | 0.000243 | -0.1032 | down |
| Proline betaine | 1.6844 | 0.008915 | -0.1145 | down |
| Acylcarnitines (C18:1) | 1.6718 | 6.33E-05 | -0.075 | down |
| Lactate | 1.6703 | 0.001837 | -0.0957 | down |
| Cyclopentanol | 1.6696 | 0.003728 | -0.0729 | down |
| N,N-Dimethylarginine | 1.6393 | 0.02197 | -0.1002 | down |
| Polidocanol | 1.6294 | 0.000126 | -0.074 | down |
| Cer (d18:1/24:0) | 1.6223 | 0.01496 | -0.1196 | down |
| N-Nervonoyl Tyrosine | 1.615 | 2.63E-05 | -0.0728 | down |
| Hypoxanthine | 1.5889 | 0.002506 | -0.0865 | down |
| Isopropyl unoprostone | 1.5858 | 0.0003 | -0.067 | down |
| 13Z-Docosenamide | 1.5819 | 2.69E-05 | -0.0648 | down |
| Xanthurenic acid | 1.5724 | 0.01272 | -0.0799 | down |
| Phenylacetic acid | 1.5714 | 0.01484 | -0.0963 | down |
| N-Methylphenylalanine | 1.5671 | 0.009161 | -0.0935 | down |
| L-Glycine | 1.5641 | 0.03186 | -0.1119 | down |
| N-Acetylneuraminic acid | 1.5625 | 0.000157 | -0.084 | down |
| Pipecolic Acid | 1.5617 | 0.008887 | -0.0844 | down |
| L-Aspartic acid | 1.5585 | 0.007362 | -0.0863 | down |
| Denzimol | 1.5568 | 0.001892 | -0.0898 | down |
| Glycerylmonooleate | 1.5565 | 4.58E-05 | -0.0656 | down |
| Fagomine | 1.5543 | 0.000181 | -0.0903 | down |
| Ligusticide | 1.5492 | 0.001221 | -0.0849 | down |
| Ribose 1-phosphate | 1.5347 | 0.04023 | 0.1649 | up |
| Adenine | 1.5345 | 0.04469 | -0.1034 | down |
| Minaxolone | 1.5332 | 0.001095 | -0.0766 | down |
| Decyl alcohol | 1.5301 | 0.03157 | -0.1048 | down |
| Dioctyl phthalate | 1.5255 | 0.000782 | -0.0567 | down |
| Xanthosine | 1.5254 | 0.02662 | -0.1057 | down |
| Malonyl-CoA | 1.5205 | 0.000236 | -0.0693 | down |
| 11-Deoxy-PGE1 | 1.5188 | 0.000414 | -0.074 | down |
| 7-Epijasmonic acid | 1.5056 | 0.005613 | -0.0795 | down |
| Phosphate | 1.5053 | 0.002548 | -0.093 | down |

VIP: variable importance in the projection; FC: fold change; CON: cows without hesperidin; HES: cows fed with hesperidin.
