# Supplementary material for: Hesperidin alleviates systemic inflammation and oxidative stress by remodeling adipose tissue lipid metabolism in periparturient dairy cows
Source: J Anim Sci Biotechnol. 2026 Apr 5;17:58. doi: 10.1186/s40104-026-01372-4 (PMC13050489; doi:10.1186/s40104-026-01372-4)
Supplement: Supplementary file 5 — Additional file 5: Fig. S4. Lipid classification in adipose tissue and serum. [file 40104_2026_1372_MOESM5_ESM.docx]

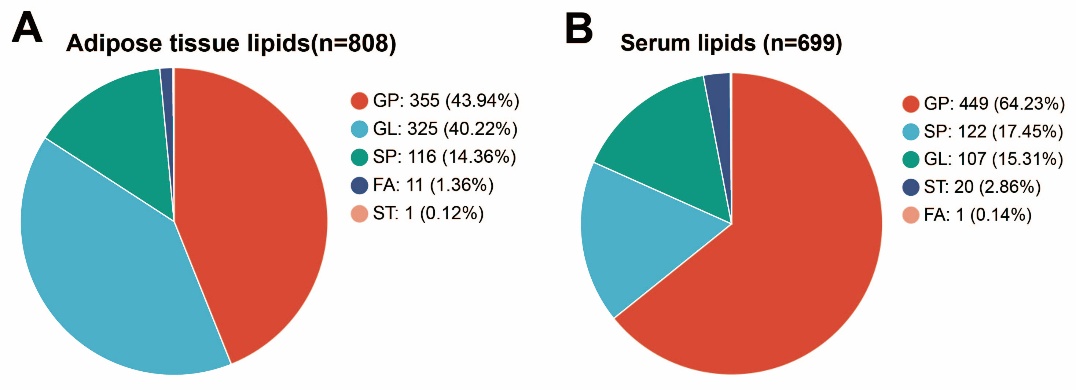


**Fig. S4.** Lipid classification in adipose tissue and serum. (A) Distribution of lipid species identified in adipose tissue (n = 808), including glycerophospholipids (GP, 43.94%), glycerolipids (GL, 40.22%), sphingolipids (SP, 14.36%), fatty acyls (FA, 1.36%), and sterol lipids (ST, 0.12%).

(B) Distribution of lipid species identified in serum (n = 699), including glycerophospholipids (GP, 64.23%), sphingolipids (SP, 17.45%), glycerolipids (GL, 15.31%), sterol lipids (ST, 2.86%), and fatty acyls (FA, 0.14%).
