# Supplementary material for: Hesperidin alleviates systemic inflammation and oxidative stress by remodeling adipose tissue lipid metabolism in periparturient dairy cows
Source: J Anim Sci Biotechnol. 2026 Apr 5;17:58. doi: 10.1186/s40104-026-01372-4 (PMC13050489; doi:10.1186/s40104-026-01372-4)
Supplement: Supplementary file 6 — Additional file 6: Table S4. Differential lipid species in adipose tissue samples between CON and HES cows. [file 40104_2026_1372_MOESM6_ESM.docx]

Table S4. Differential lipid species in adipose tissue samples between CON and HES cows.

| Lipid species | Category | Class | VIP | P-value | Log2(FC) | Regulate |
| --- | --- | --- | --- | --- | --- | --- |
| PE(18:0e/18:1) | GP | PE | 1.7983 | 0.01692 | 0.056029 | up |
| PE(18:0e/18:2) | GP | PE | 1.8707 | 0.02469 | 0.058247 | up |
| PE(16:0e/18:2) | GP | PE | 1.8298 | 0.04306 | 0.057693 | up |
| PE(16:0e/18:1) | GP | PE | 1.7578 | 0.0274 | 0.051859 | up |
| PS(16:0/18:1) | GP | PS | 1.6019 | 0.03285 | 0.06226 | up |
| PS(18:1/18:2) | GP | PS | 1.852 | 0.005722 | 0.074916 | up |
| PS(16:0/18:2) | GP | PS | 2.0522 | 0.008601 | 0.085968 | up |
| Cer(d18:1/16:0) | SP | Cer | 2 | 0.006363 | -0.06462 | down |
| PS(20:2e/18:1) | GP | PS | 1.5817 | 0.04327 | 0.051163 | up |
| Cer(d18:1/18:0) | SP | Cer | 1.9371 | 0.00731 | -0.0687 | down |
| PE(14:1e/18:2) | GP | PE | 1.6448 | 0.04013 | 0.045443 | up |
| Cer(d18:1/24:0) | SP | Cer | 2.0417 | 0.005684 | -0.07476 | down |
| PS(16:0/20:4) | GP | PS | 2.1394 | 0.003962 | 0.094777 | up |
| Hex2Cer(d16:0/24:1) | SP | Hex2Cer | 1.5642 | 0.001305 | 0.040822 | up |
| DG(19:0/18:1) | GL | DG | 2.114 | 0.002507 | -0.06991 | down |
| DG(18:1/18:3) | GL | DG | 2.1564 | 0.002713 | -0.07795 | down |
| PS(18:2e/18:1) | GP | PS | 1.6643 | 0.03588 | 0.061154 | up |
| Hex2Cer(d16:0/26:1) | SP | Hex2Cer | 1.7231 | 0.001142 | 0.057277 | up |
| PI(16:0/18:2) | GP | PI | 1.7868 | 0.01025 | 0.068877 | up |
| SM(d20:0/24:2) | SP | SM | 1.8987 | 0.02101 | 0.06929 | up |
| LPC(16:0) | GP | LPC | 1.5393 | 0.04121 | -0.06341 | down |
| PS(20:2e/18:2) | GP | PS | 1.8851 | 0.01872 | 0.067501 | up |
| PS(17:0/18:2) | GP | PS | 1.9365 | 0.008649 | 0.098015 | up |
| PS(18:2/20:4) | GP | PS | 2.2508 | 0.01172 | 0.132643 | up |
| Hex2Cer(d16:0/26:2) | SP | Hex2Cer | 2.7927 | 0.000487 | 0.152378 | up |
| MGDG(14:1e/24:0) | GL | MGDG | 1.6839 | 0.02398 | 0.045163 | up |
| PC(12:1e/18:2) | GP | PC | 1.6353 | 0.04152 | 0.045024 | up |
| PC(30:0/18:1) | GP | PC | 2.442 | 0.01166 | 0.139339 | up |
| LPE(18:1) | GP | LPE | 1.6831 | 0.001945 | -0.06416 | down |
| PE(15:0/18:2) | GP | PE | 1.7011 | 0.03611 | 0.086376 | up |
| TG(15:0/8:0/16:0) | GL | TG | 1.8491 | 0.004329 | -0.06341 | down |
| PE(18:0e/17:1) | GP | PE | 1.8353 | 0.02182 | 0.065986 | up |
| PS(16:0/16:1) | GP | PS | 2.3141 | 0.003581 | 0.137766 | up |
| SM(d17:1/16:0) | SP | SM | 1.533 | 0.002283 | 0.035202 | up |
| PE(18:0p/20:3) | GP | PE | 1.5019 | 0.04137 | 0.034216 | up |
| SM(d17:0/18:1) | SP | SM | 1.8336 | 0.002151 | 0.044884 | up |
| PI(16:0/18:1) | GP | PI | 1.6351 | 0.008163 | 0.035061 | up |
| CL(18:2/16:0/20:3/20:3) | GP | CL | 1.6045 | 0.01257 | 0.046701 | up |
| SM(d18:1/24:2) | SP | SM | 2.4462 | 0.005188 | 0.066675 | up |
| CL(20:4/16:0/20:4/24:1) | GP | CL | 1.6562 | 0.009325 | 0.048934 | up |
| PC(18:1e/18:2) | GP | PC | 1.5653 | 0.01837 | 0.035624 | up |
| PC(16:1e/18:2) | GP | PC | 1.5827 | 0.01641 | 0.035624 | up |
| PC(18:0e/16:0) | GP | PC | 1.8594 | 0.01301 | 0.048515 | up |
| PC(20:4e/15:0) | GP | PC | 1.7312 | 0.02121 | 0.045303 | up |
| SM(d18:1/16:0) | SP | SM | 1.5262 | 0.003083 | 0.035905 | up |
| PC(18:3e/15:0) | GP | PC | 1.9685 | 0.04802 | 0.050328 | up |
| SM(d18:2/16:0) | SP | SM | 1.5273 | 0.005476 | 0.042084 | up |
| SM(d18:1/22:0) | SP | SM | 1.5364 | 0.007675 | 0.033652 | up |
| SM(d18:1/24:0) | SP | SM | 1.8101 | 0.005206 | 0.039981 | up |
| PC(14:0e/20:4) | GP | PC | 1.7036 | 0.02107 | 0.036749 | up |
| PC(16:1/19:0) | GP | PC | 1.6904 | 0.000954 | 0.041243 | up |
| PC(20:4e/17:0) | GP | PC | 1.7016 | 0.000332 | 0.045303 | up |
| SM(d18:1/24:1) | SP | SM | 1.8869 | 0.004935 | 0.043064 | up |
| SM(d18:1/22:1) | SP | SM | 2.165 | 0.009492 | 0.055057 | up |
| CL(18:2/18:1/18:2/18:2) | GP | CL | 1.716 | 0.005713 | 0.051442 | up |
| PC(16:2e/17:0) | GP | PC | 1.7009 | 0.01466 | 0.047259 | up |
| PC(18:0e/18:2) | GP | PC | 2.4837 | 0.003825 | 0.076969 | up |
| TG(16:1/14:1/18:3) | GL | TG | 1.528 | 0.009921 | 0.038155 | up |
| SM(d19:0/24:2) | SP | SM | 1.6232 | 0.001554 | 0.040402 | up |
| PE(18:1p/18:2) | GP | PE | 1.9267 | 0.01236 | 0.04712 | up |
| TG(18:1/18:1/22:4) | GL | TG | 1.6659 | 0.007279 | 0.045583 | up |
| PC(14:1e/18:2) | GP | PC | 2.1284 | 0.02162 | 0.059909 | up |
| PC(18:3e/17:0) | GP | PC | 1.7292 | 0.00737 | 0.047818 | up |
| PC(15:0/18:3) | GP | PC | 1.8216 | 0.008991 | 0.04991 | up |
| PE(18:0p/18:2) | GP | PE | 1.7041 | 0.01481 | 0.036468 | up |
| SM(d18:0/22:0) | SP | SM | 1.6489 | 0.008137 | 0.040963 | up |
| SM(d14:0/18:0) | SP | SM | 1.5357 | 0.007604 | 0.042644 | up |
| PC(16:2e/19:0) | GP | PC | 1.6491 | 0.001534 | 0.04991 | up |
| PI(18:1/18:2) | GP | PI | 1.5804 | 0.01477 | 0.048236 | up |
| PC(16:2e/18:1) | GP | PC | 1.6967 | 0.01892 | 0.04698 | up |
| PC(18:0e/18:1) | GP | PC | 2.0548 | 0.01437 | 0.069152 | up |
| Hex2Cer(d18:1/16:0) | SP | Hex2Cer | 2.6508 | 0.000711 | 0.102188 | up |
| PE(18:1p/20:3) | GP | PE | 1.5514 | 0.002494 | 0.047678 | up |
| PI(18:1/20:4) | GP | PI | 1.7203 | 0.003915 | 0.056306 | up |
| LPC(18:0) | ST | LPC | 1.6861 | 0.01831 | -0.06613 | down |
| SM(d20:0/24:3) | SP | SM | 2.8438 | 0.004944 | 0.114234 | up |
| PI(16:0/20:3) | GP | PI | 1.726 | 0.04596 | 0.051302 | up |
| PI(18:0/20:4) | GP | PI | 1.892 | 0.01008 | 0.069565 | up |
| MePC(12:0e/18:2) | GP | MePC | 1.6618 | 0.01443 | 0.053389 | up |
| PC(8:0/11:1) | GP | PC | 1.6408 | 0.009344 | 0.051859 | up |
| PE(14:0p/18:2) | GP | PE | 2.1436 | 0.01338 | 0.069427 | up |
| PI(18:0/20:5) | GP | PI | 1.649 | 0.01327 | 0.055751 | up |
| Cer(m18:1/24:0) | SP | Cer | 1.6098 | 0.01277 | -0.03964 | down |
| Hex2Cer(d18:1/24:1) | SP | Hex2Cer | 3.6488 | 0.000746 | 0.215616 | up |
| PC(8:0/11:2) | GP | PC | 2.0051 | 0.01001 | 0.060877 | up |
| PC(16:1/13:0) | GP | PC | 1.5837 | 0.02438 | 0.066261 | up |
| PE(14:0p/20:3) | GP | PE | 2.2791 | 0.01759 | 0.074916 | up |
| SM(d18:1/12:0) | SP | SM | 1.8371 | 0.001957 | 0.055751 | up |
| SM(d17:0/16:0) | SP | SM | 1.7897 | 0.01858 | 0.04698 | up |
| SM(d18:0/24:0) | SP | SM | 1.8802 | 0.003285 | 0.054501 | up |
| SM(d18:1/24:4) | SP | SM | 2.1752 | 0.002553 | 0.066675 | up |
| SM(d19:0/24:3) | SP | SM | 2.6791 | 0.004414 | 0.098958 | up |
| TG(18:0/18:0/18:0) | GL | TG | 2.4249 | 0.01812 | -0.10129 | down |

VIP: variable importance in the projection; FC: fold change; CON: cows without hesperidin; HES: cows fed with hesperidin.
