# Supplementary material for: Hesperidin alleviates systemic inflammation and oxidative stress by remodeling adipose tissue lipid metabolism in periparturient dairy cows
Source: J Anim Sci Biotechnol. 2026 Apr 5;17:58. doi: 10.1186/s40104-026-01372-4 (PMC13050489; doi:10.1186/s40104-026-01372-4)
Supplement: Supplementary file 7 — Additional file 7: Fig. S5. Proteomic overview of adipose tissue in control (CON) and hesperidin (HES) cows. [file 40104_2026_1372_MOESM7_ESM.docx]

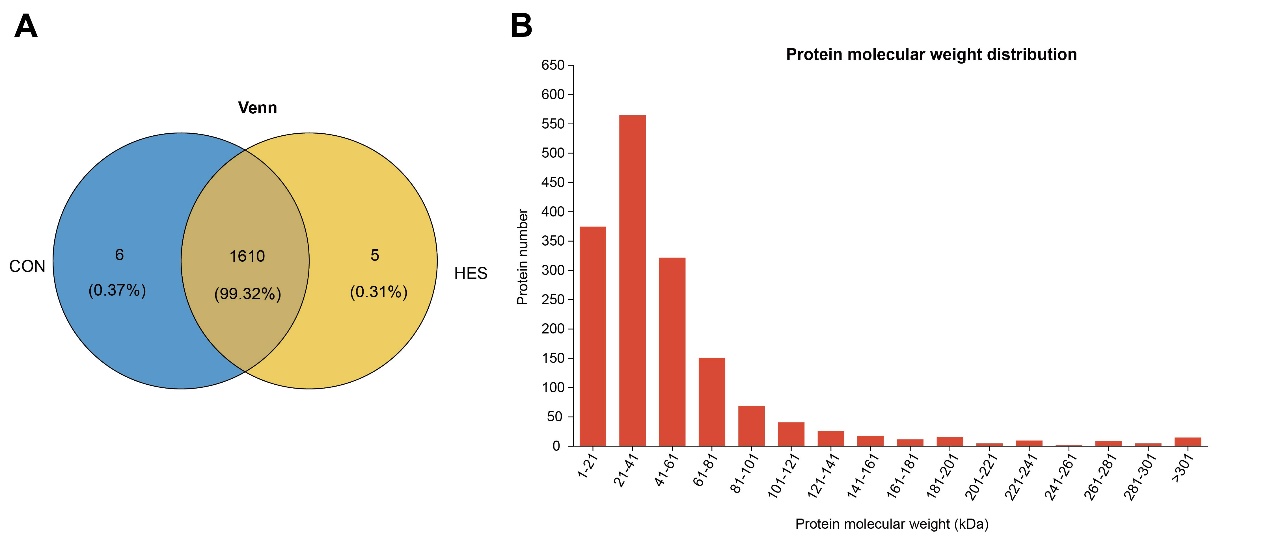


**Fig. S5.** Proteomic overview of adipose tissue in control (CON) and hesperidin (HES) cows.

(A) Venn diagram showing the overlap of identified proteins between CON and HES groups. A total of 1,610 proteins (99.32%) were commonly detected, with 6 and 5 unique proteins identified in CON and HES, respectively. (B) Distribution of identified proteins according to molecular weight. The majority of proteins fell within the 1–60 kDa range, with fewer proteins detected at higher molecular weights (>100 kDa).
