# Supplementary material for: Hesperidin alleviates systemic inflammation and oxidative stress by remodeling adipose tissue lipid metabolism in periparturient dairy cows
Source: J Anim Sci Biotechnol. 2026 Apr 5;17:58. doi: 10.1186/s40104-026-01372-4 (PMC13050489; doi:10.1186/s40104-026-01372-4)
Supplement: Supplementary file 9 — Additional file 9: Table S6. Differential lipid species in serum samples between CON and HES cows. [file 40104_2026_1372_MOESM9_ESM.docx]

Table S6. Differential lipid species in serum samples between CON and HES cows.

| Lipid species | Category | Class | VIP | P-value | Log2(FC) | Regulate |
| --- | --- | --- | --- | --- | --- | --- |
| PG(16:1e/20:4) | GP | PG | 7.605 | 0.03035 | 1.20364 | up |
| Cer(t17:0/23:0) | SP | Cer | 2.8509 | 0.04781 | -0.08421 | down |
| PE(16:1e/22:4) | GP | PE | 2.7427 | 0.04556 | 0.100305 | up |
| Cer(d18:0/22:0) | SP | Cer | 2.5347 | 0.01484 | -0.05035 | down |
| PC(14:1e/18:2) | GP | PC | 2.3784 | 0.00347 | 0.037031 | up |
| MePC(14:0e/18:2) | GP | MePC | 2.3541 | 0.000282 | 0.031113 | up |
| SM(d18:0/22:0) | SP | SM | 2.3149 | 0.02425 | 0.038998 | up |
| PC(18:3e/24:2) | GP | PC | 2.309 | 0.02129 | 0.046282 | up |
| PE(18:1p/18:2) | GP | PE | 2.236 | 0.02034 | 0.045303 | up |
| PC(20:0e/20:4) | GP | PC | 2.1932 | 0.01041 | 0.035342 | up |
| LPC(16:0) | GP | LPC | 2.1625 | 0.02027 | -0.03239 | down |
| PC(20:4e/19:1) | GP | PC | 2.1361 | 0.01237 | 0.029559 | up |
| PC(14:1e/20:4) | GP | PC | 2.0643 | 0.006221 | 0.034075 | up |
| PS(20:1e/22:4) | GP | PS | 2.0074 | 0.03012 | 0.067363 | up |
| SM(d18:1/24:2) | SP | SM | 1.9813 | 0.004539 | 0.029135 | up |
| PC(16:1e/20:4) | GP | PC | 1.9755 | 0.02602 | 0.033793 | up |
| MePC(16:1e/18:2) | GP | MePC | 1.9675 | 0.03549 | 0.030972 | up |
| CL(21:0/16:0/16:0/20:4) | GP | CL | 1.8902 | 0.01222 | 0.040402 | up |
| PS(18:0e/22:4) | GP | PS | 1.8503 | 0.01191 | 0.050188 | up |
| PC(20:4e/17:1) | GP | PC | 1.8282 | 0.000451 | 0.018634 | up |
| PC(18:3e/20:3) | GP | PC | 1.8254 | 0.001683 | 0.017637 | up |
| PS(20:0e/18:2) | GP | PS | 1.8141 | 0.003049 | 0.026588 | up |
| PS(20:0e/20:4) | GP | PS | 1.7741 | 6.44E-05 | 0.030265 | up |
| PS(20:1e/18:2) | GP | PS | 1.7483 | 0.02366 | 0.036749 | up |
| PC(18:3e/15:0) | GP | PC | 1.7469 | 0.02547 | 0.02219 | up |
| TG(18:0/16:0/18:1) | GL | TG | 1.7436 | 0.01364 | -0.018 | down |
| Cer(d18:1/16:0) | SP | Cer | 1.7342 | 0.013 | -0.04038 | down |
| PS(20:1e/20:4) | GP | PS | 1.7331 | 0.01085 | 0.035765 | up |
| PS(20:1e/18:1) | GP | PS | 1.7277 | 0.01267 | 0.033793 | up |
| SM(d18:1/24:4) | SP | SM | 1.7269 | 0.000253 | 0.03196 | up |
| PC(18:3e/17:0) | GP | PC | 1.7027 | 0.02453 | 0.023468 | up |
| SM(d20:0/24:3) | SP | SM | 1.679 | 0.0382 | 0.028993 | up |
| PS(20:0e/22:4) | GP | PS | 1.6731 | 0.02053 | 0.038296 | up |
| TG(16:0/18:1/24:0) | GL | TG | 1.6577 | 0.03114 | -0.02518 | down |
| PC(18:2e/17:0) | GP | PC | 1.6567 | 0.01698 | 0.017922 | up |
| PE(18:2e/18:0) | GP | PE | 1.6267 | 0.009176 | 0.026163 | up |
| PS(18:0e/22:5) | GP | PS | 1.6203 | 0.0451 | 0.044044 | up |
| PS(18:0e/18:1) | GP | PS | 1.6197 | 0.02163 | 0.029842 | up |
| PC(16:1e/17:1) | GP | PC | 1.6197 | 0.02163 | 0.029842 | up |
| SM(d18:1/24:0) | SP | SM | 1.6149 | 0.01185 | 0.044044 | up |
| TG(16:0/18:1/23:0) | GL | TG | 1.6103 | 0.02521 | -0.02532 | down |
| Cer(d18:1/24:0) | SP | Cer | 1.6102 | 0.004532 | -0.02342 | down |
| LPC(18:0) | GP | LPC | 1.5947 | 0.0275 | -0.02635 | down |
| Cer(d18:1/18:0) | SP | Cer | 1.5833 | 0.02699 | -0.0265 | down |
| PC(18:0e/22:4) | GP | PC | 1.575 | 0.01208 | 0.042364 | up |
| PS(18:0e/16:0) | GP | PS | 1.5739 | 0.02668 | 0.022048 | up |
| PS(20:0e/22:5) | GP | PS | 1.5188 | 0.034 | 0.039138 | up |

VIP: variable importance in the projection; FC: fold change; CON: cows without hesperidin; HES: cows fed with hesperidin.
